# Overcoming immunogenic gaps in malaria subunit vaccines by broadening CSP regions targeted

**DOI:** 10.1084/jem.20260846

**Published:** 2026-08-07

**Authors:** Ja-Hyun Koo, Prabhanshu Tripathi, Yevel Flores-Garcia, Kazutoyo Miura, Ganchimeg Bayarsaikhan, Chen-Hsiang Shen, Stephanie R. Weldon, Sven Kratochvil, Thavaleak Prum, Ali A. Albowaidey, Ye-Ji Kim, Jordan R. Ellis-Pugh, Quynh Anh Phan, Jennifer Suurbaar, Robert Sifa Onjiko, Misook Choe, Gordon A. Dale, Haotian Lei, Nicholas C. Morano, Lais Da Silva Pereira, Marlon Dillon, Michael F. Bender, Mariah Lofgren, Baoshan Zhang, Theodore C. Pierson, Lawrence Shapiro, Tongqing Zhou, Usha Nair, Azza H. Idris, Fidel Zavala, Peter D. Kwong, Robert A. Seder, Facundo D. Batista

**Affiliations:** 1 Batista Lab, The Ragon Institute of Mass General Brigham, MIT, and Harvard, Cambridge, MA, USA; 2 Vaccine Research Center, National Institutes of Health, Bethesda, MD, USA; 3 Department of Molecular Microbiology and Immunology, Johns Hopkins Bloomberg School of Public Health, Baltimore, MD, USA; 4 Idris Lab, The Ragon Institute of Mass General Brigham, MIT, and Harvard, Cambridge, MA, USA; 5 Research Technologies Branch, National Institute of Allergy and Infectious Diseases, National Institutes of Health, Bethesda, MD, USA; 6 Aaron Diamond AIDS Research Center and Department of Biochemistry, Columbia University Vagelos College of Physicians and Surgeons, New York, NY, USA; 7 Department of Pediatrics, Harvard Medical School, Boston, MA, USA; 8 Department of Biology, Massachusetts Institute of Technology, Cambridge, MA, USA; 9 Department of Immunology, Harvard Medical School, Boston, MA, USA

## Abstract

The first approval of malaria vaccines RTS,S/AS01 and R21/Matrix M marked a major milestone. Both vaccines present a portion of the *Plasmodium falciparum* circumsporozoite protein (PfCSP) containing an immunodominant major repeat region, which may limit breadth to other protective epitopes, limiting efficacy and durability. Using B cell receptor (BCR) knock-in mice, we found that the R21-included PfCSP epitope elicited robust B cell responses to the immunodominant major repeats but not to other highly protective epitopes, the minor repeat and junction. We then defined a minimal peptide capable of eliciting minor repeat–specific B cell responses and generating highly protective antibodies (Abs). We characterized these Abs bioinformatically and structurally to identify protective traits, which informed the design of variant Abs with improved affinity. Finally, we demonstrated that vaccination combining the R21-included PfCSP epitope, the minimal minor repeat peptide, and a junctional region immunogen elicited balanced B cell and Ab responses, enhancing protection in vivo. Broadening responses through immunofocusing may overcome immunogenic gaps in current malaria vaccines.

## Teaser

Koo et al. demonstrate that *Plasmodium falciparum* circumsporozoite major repeat epitope immunodominance inhibits other highly protective epitopes; current vaccines present this major repeat epitope. The authors show that adding peptide immunogens overcomes this immunogenic gap and enhances protection in vivo.

## Graphical Abstract

**Figure:**
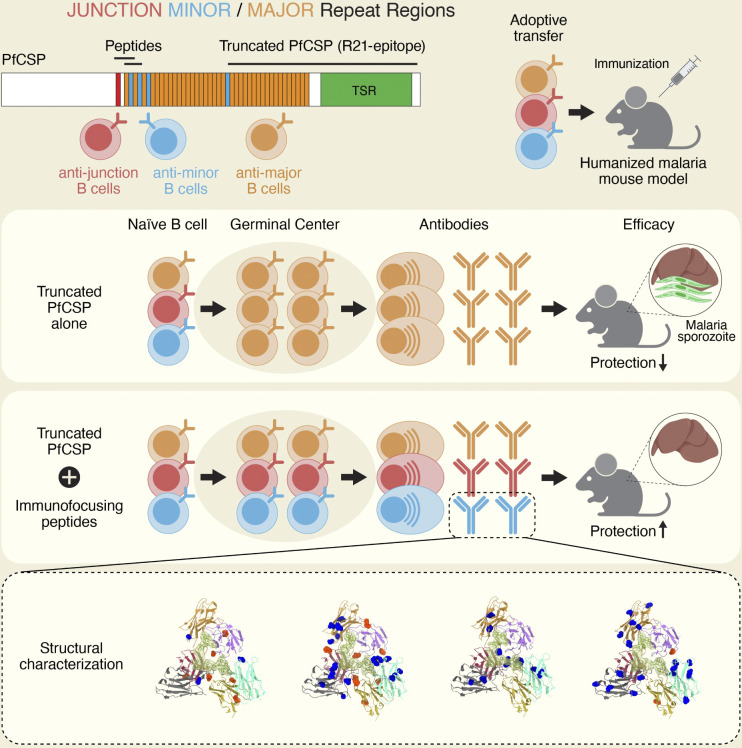

## Introduction

Malaria caused by infection with *Plasmodium falciparum* leads to more than half a million deaths a year, primarily in young children in Africa (World Health Organization, 2024). Infection is initiated by a mosquito bite; the plasmodial parasite enters through the dermis or blood as a small number of sporozoites (SPZ) (Frischknecht and Matuschewski, 2017; Phillips et al., 2017). SPZ then quickly reach the liver to exponentially expand before release into the blood and clinical infection. Prevention efforts have focused on limiting SPZ infection in the liver (Cockburn and Seder, 2018).

The World Health Organization has recommended two vaccines for malaria prevention, RTS,S and R21 (World Health Organization, 2024). Both are targeted to the *P*. *falciparum* circumsporozoite protein (CSP), which covers the exterior of the SPZ (Nussenzweig and Nussenzweig, 1985) and is composed of N-terminal, central repeat, and C-terminal domains. The central repeat domain primarily consists of ∼40 repeats of the amino acids (AAs) NANP, also known as the major repeats, as well as one to four repeats of NVDP, known as the minor repeat region (Enea et al., 1984; Zeeshan et al., 2012). Additionally, there is a junctional region that connects the central repeat domain to the N-terminal domain. Both RTS,S and R21 vaccines present a portion of the NANP central repeat region and the C terminus fused to a hepatitis B surface antigen (HBsAg) (Gordon et al., 1995). RTS,S provides ∼50% protection at 1 year and 36% protection at 4 years (Feng et al., 2022; RTS,S Clinical Trials Partnership, 2015). R21 has ∼75% efficacy after 1 year (Datoo et al., 2024). Efficacy for both RTS,S and R21 requires an initial three-dose series in the first year of life and a booster in the second year of life to maintain high titers of protective antibodies (Abs) (Datoo et al., 2024). The requirement for multiple doses over 2 years and the waning efficacy over time highlight the importance of maintaining a magnitude of Ab responses sufficient to limit infection (White et al., 2015). The induction of higher potency Abs at lower titers or increasing the breadth of Ab responses to other regions across *P*. *falciparum* CSP (PfCSP) not contained in the current vaccines provides a potentially promising approach to enhance the efficacy and durability of protection by PfCSP-based vaccines.

Recently, the discovery of mAbs against the junctional and minor repeat regions of PfCSP that are highly effective at preventing malaria infection in humans in the field has validated the importance of targeting these epitopes with vaccines (Kisalu et al., 2018; Wang et al., 2020). Neither of these epitopes is included in RTS,S or R21 (Datoo et al., 2024; Julien and Wardemann, 2019; Tan et al., 2019); specifically, both vaccines display a truncated portion of PfCSP containing 18 central NANP repeats and the C-terminal region (Collins et al., 2017). Recent human clinical trials have demonstrated the high protective efficacy of the anti-junctional CIS43LS and anti-minor L9LS Abs (Wu et al., 2022; Kayentao et al., 2022, 2024; Gaudinski et al., 2021), underscoring the importance of eliciting responses against these epitopes. Both the junctional and minor epitopes contain NANP sequences, highlighting promiscuity in Abs—including L9 and CIS43—that may bind across the major, minor, and junctional regions (Wang et al., 2024; Julien and Wardemann, 2019); Abs capable of binding these regions may, therefore, be elicited by R21 and RTS,S. However, our group previously found that immunofocusing to the junctional region, rather than delivering the full PfCSP protein, was a more effective method for the elicitation of anti-junction responses (Kratochvil et al., 2021); alternative deliveries of junctional epitopes have also demonstrated some protective efficacy (Francica et al., 2021; Jelínková et al., 2021; Tripathi et al., 2026). The efficacy of current vaccines in eliciting responses to these regions remains unknown.

Here, we used a set of transgenic mouse models with B cells expressing mature or germline equivalents of human antimalarial Abs to recapitulate the development of protective responses by these direct specificities of the human B cell repertoire. We assessed the specificity of B cell and Ab responses using immunogens to the major, minor, and junctional regions alone or in combination. We first show that neither the portions of PfCSP included in the R21 vaccine nor full-length PfCSP could activate anti-minor precursor B cells of the highly protective mAb L9 (Wang et al., 2020). To address this limitation, we identified an anti-minor repeat immunofocusing peptide that successfully activated these precursor B cells. This peptide induced longer germinal center (GC) responses than the full-length PfCSP protein, enabling extended somatic hypermutation (SHM), and also promoted significant GC responses and further affinity maturation of high-affinity mature L9. We used bioinformatic, biophysical, and structural analyses to characterize the Ab responses after immunization. Furthermore, combining PfCSP epitopes present in R21 with the immunofocusing peptides elicited balanced and protective B cell and Ab responses to major, minor, and junctional epitopes, offering a promising strategy to enhance malaria vaccine efficacy.

## Results

### Breadth of PfCSP-specific B cell and Ab responses following vaccination with an R21-epitope immunogen

**Figure 1.**
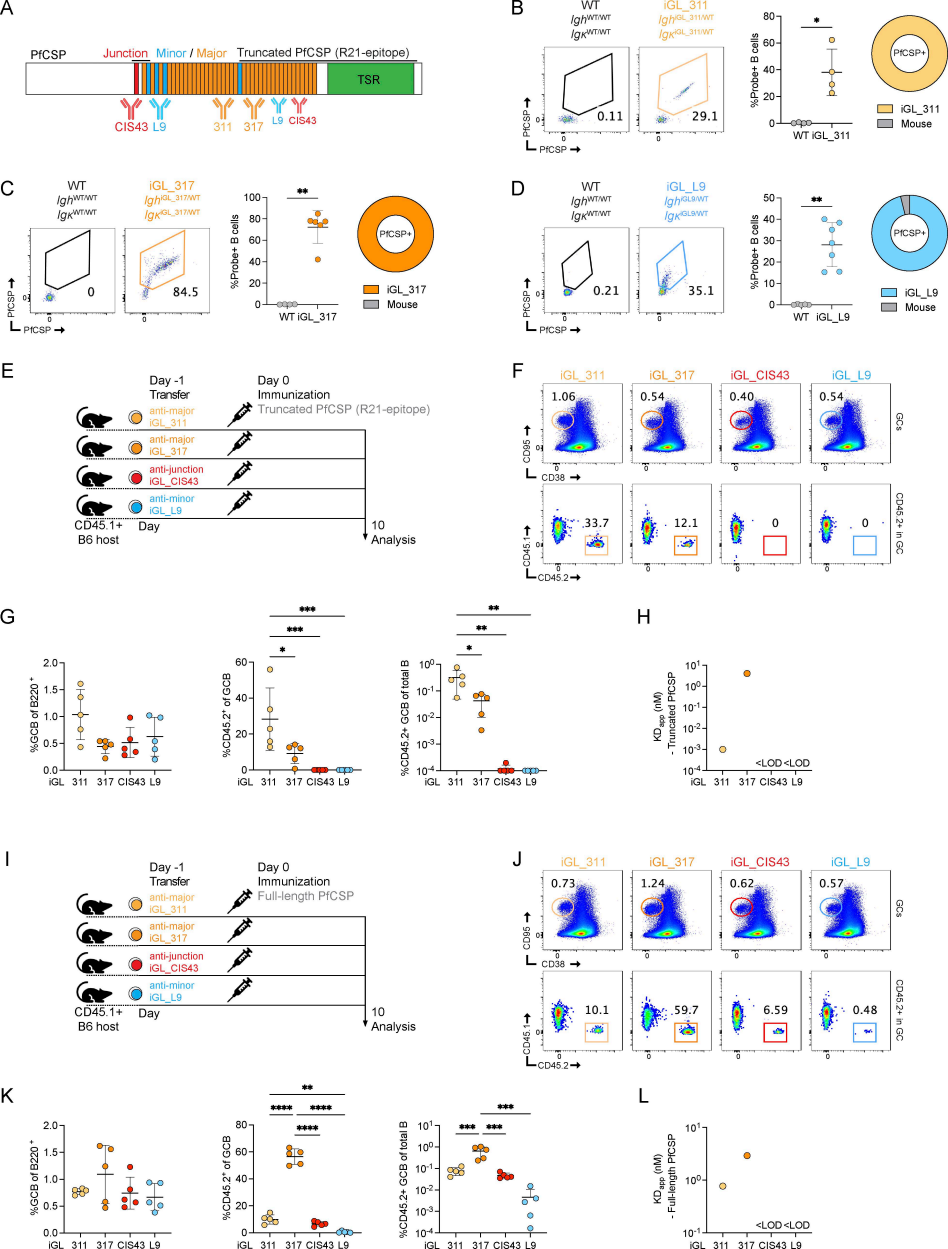
**Response of anti-CSP Ab precursor B cells to immunization by an R21-epitope immunogen. (A)** Schematic of PfCSP and antimalaria Abs (Kratochvil et al., 2021). mAbs 311 and 317 primarily target the major repeat region of PfCSP. CIS43 and L9 primarily target the junctional or minor repeat regions but also exhibit affinity for the major repeat region. “Truncated PfCSP” describes the epitope presented by R21. **(B)** Left: Representative FACS plots showing PfCSP binding of peripheral blood B cells isolated from WT C57BL/6J or 311 precursor (iGL_311) BCR KI mouse model (*Igh*^*iGL_317/WT*^*, Igκ*^*iGL_311/WT*^). Center: Quantification of PfCSP binding of peripheral blood B cells from the iGL_311 mouse model, *N* = 4. The data are pooled from two independent experiments. Right: Sequence analysis of sorted total B cells or PfCSP^+^ B cells from the iGL_311 mouse model. Yellow indicates B cells with paired iGL_311 HC and LC sequences, while gray indicates B cells with paired HC and LC sequences of endogenous mouse origin. Total of 30 paired sequences from one mouse. Mann–Whitney’s *t* test was applied, and the bars indicate mean ± SD. *P < 0.05. **(C)** Left: Representative FACS plots showing PfCSP binding of peripheral blood B cells isolated from WT C57BL/6J or 317 precursor (iGL_317) BCR KI mouse model (*Igh*^*iGL_317/WT*^*, Igκ*^*iGL_317/WT*^). Center: Quantification of PfCSP binding of peripheral blood B cells from the iGL_317 mouse model, *N* = 6. The data are pooled from two independent experiments. Right: Sequence analysis of sorted total B cells or PfCSP^+^ B cells from the iGL_317 mouse model. Orange indicates B cells with paired iGL_317 HC and LC sequences, while gray indicates B cells with paired HC and LC sequences of endogenous mouse origin. Total of 23 paired sequences from one mouse. Mann–Whitney’s *t* test was applied, and the bars indicate mean ± SD. **P < 0.01. **(D)** Left: Representative FACS plots showing PfCSP binding of peripheral blood B cells isolated from WT C57BL/6J or L9 precursor (iGL_L9) BCR KI mouse model (*Igh*^*iGL_L9/WT*^*, Igκ*^*iGL_L9/WT*^). Center: Quantification of PfCSP binding of peripheral blood B cells from the iGL_L9 mouse model, *N* = 7. The data are pooled from two independent experiments. Right: Sequence analysis of sorted total B cells or PfCSP^+^ B cells from the iGL_L9 mouse model. Light blue indicates B cells with paired iGL_L9 HC and LC sequences, while gray indicates B cells with paired HC and LC sequences of endogenous mouse origin. Total of 51 paired sequences from two mice. Mann–Whitney’s *t* test was applied, and the bars indicate mean ± SD. **P < 0.01. **(E)** Schematic of adoptive transfer model to evaluate immune response to truncated PfCSP (R21-epitope). B cells isolated from each BCR KI mouse (CD45.2) were transferred into CD45.1 host mice at a precursor frequency of 5 per 10^5^ cells on day −1, followed by intraperitoneal immunization with truncated PfCSP on day 0. Splenic B cell responses were analyzed on day 10. **(F)** Representative FACS plots showing (top) GC (CD95^+^CD38^−^) and (bottom) CD45.2^+^ response of transfer models on day 10 after immunization. **(G)** Quantification of (left) GC B cells, (center) CD45.2^+^ in GC, and (right) CD45.2^+^ GCB frequency. *N* = 5 mice in each group from one experiment. Each symbol represents a different mouse. One-way ANOVA was applied, and the bars indicate mean ± SD. P > 0.05 was unmarked and not significant (ns), *P < 0.05, **P < 0.01, and ***P < 0.001. **(H)** Apparent affinity of anti-PfCSP germline Abs to the truncated PfCSP (R21-epitope) protein measured by BLI. <LOD indicates “below the limit of detection.” **(I)** Schematic of adoptive transfer model to evaluate immune responses by full-length PfCSP. B cells isolated from each BCR KI mouse (CD45.2) were transferred into CD45.1 host mice at a precursor frequency of 5 per 10^5^ cells on day −1, followed by intraperitoneal immunization with full-length PfCSP on day 0. Splenic B cell responses were analyzed on day 10. **(J)** Representative FACS plots showing (top) GC (CD95^+^CD38^−^) and (bottom) CD45.2^+^ response of transfer models on day 10 after immunization. **(K)** Quantification of (left) GC B cells, (center) CD45.2^+^ in GC, and (right) CD45.2^+^ GCB frequency. *N* = 5 mice in each group from one experiment. Each symbol represents a different mouse. One-way ANOVA was applied, and the bars indicate mean ± SD. P > 0.05 ns, **P < 0.01, ***P < 0.001, and ****P < 0.0001. **(L)** Apparent affinity of anti-PfCSP germline Abs to the full-length PfCSP protein measured by BLI. <LOD indicates “below the limit of detection.”

To investigate B cell responses to regions across PfCSP (Fig. 1 A), we created new knock-in (KI) B cell receptor (BCR) mouse models, with B cells bearing the pre-rearranged heavy chain (HC) and light chain (LC) sequences of the inferred germline sequences (iGL) of human anti-malarial Abs to different regions of PfCSP. We generated three new lines using our CRISPR/Cas9 approach (Lin et al., 2018; Wang et al., 2021b): two bearing inferred germline precursors to the anti-major repeat region Abs 311 and 317 (Oyen et al., 2017), iGL_311 and iGL_317, and one containing the anti-minor repeat region Ab L9 (Wang et al., 2020) precursor, iGL_L9. Additionally, we used a previously constructed KI with B cells bearing the anti-junctional region CIS43 (Kisalu et al., 2018) precursor, iGL_CIS43 (Kratochvil et al., 2021) (Fig. 1 A). We isolated B cells from each new line to confirm BCR functionality. Naïve B cells from the iGL_311 and iGL_317 models could bind to a full-length PfCSP probe, and all of the PfCSP-binding B cells bore the HC and LC sequences of iGL_311 or iGL_317 (Fig. 1, B and C). Similarly, naïve B cells from the iGL_L9 model also bound to a PfCSP probe; 96% of those PfCSP^+^ binders expressed the iGL_L9 HC and LC sequences (Fig. 1 D).

**Figure S1.**
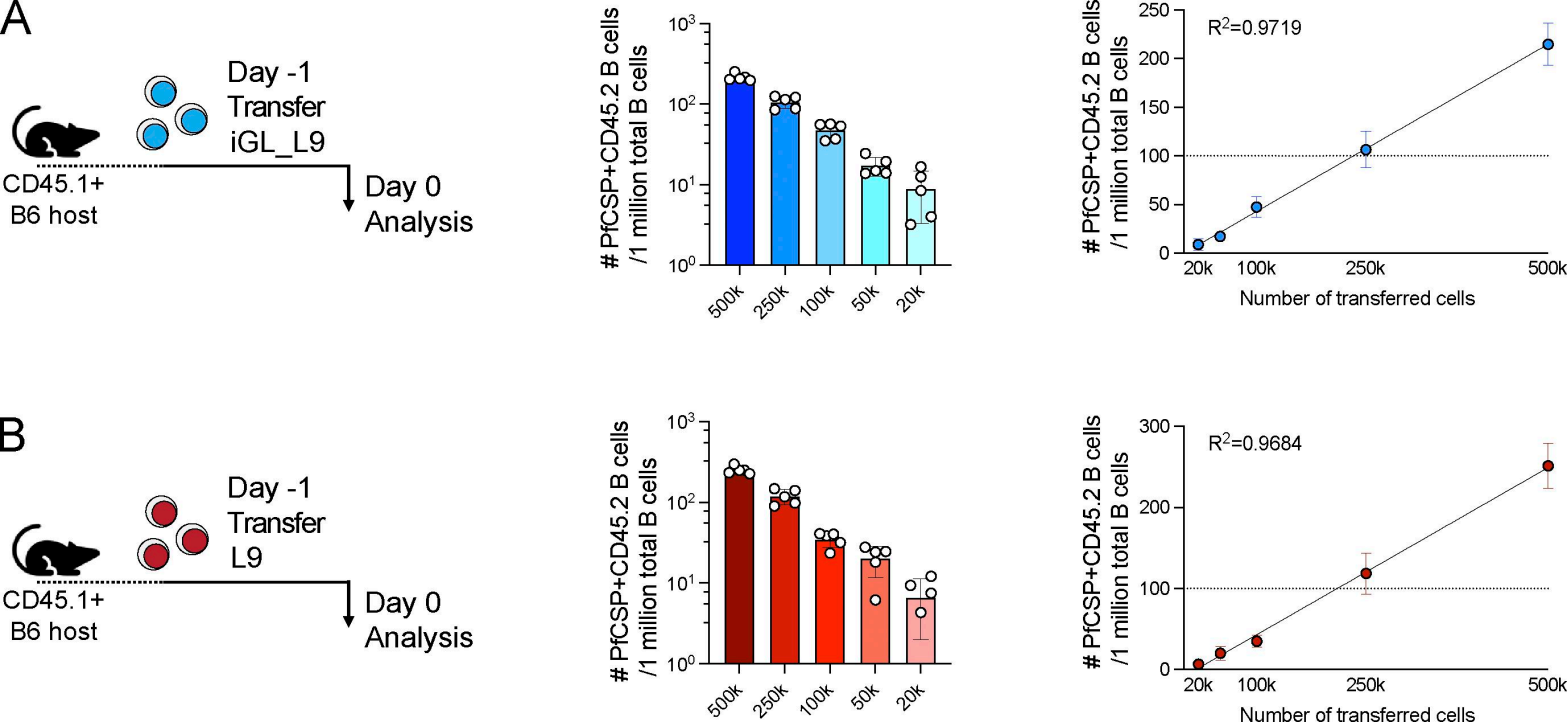
**Precursor frequency of adoptively transferred CD45.2 B cells in CD45.1 host mice, related to Fig. 1. (A)** Schematic of adoptive transfer model and quantification of frequency of transferred iGL_L9 CD45.2^+^ B cells in the CD45.1^+^ host mouse. *N* = 5 mice in each group from one experiment. R^2^ was calculated by simple linear regression. **(B)** Schematic of adoptive transfer model and quantification of frequency of transferred L9 CD45.2^+^ B cells in the CD45.1^+^ host mouse. *N* = 5 mice in each group from one experiment. R^2^ was calculated by simple linear regression.

To develop models with lower precursor frequencies more relevant to human physiology, we adoptively transferred CD45.2^+^ KI B cells intravenously into congenic CD45.1^+^ WT host mice (Abbott et al., 2018; Dosenovic et al., 2018). To estimate transfer efficiency, we transferred 20,000, 50,000, 100,000, 250,000, and 500,000 CD45.2^+^ B cells and assessed the frequency of PfCSP^+^ CD45.2^+^ cells in the spleen after 1 day (Fig. S1). While exact precursor frequencies for these specificities in humans have not yet been defined, adoptive transfer allowed us to achieve low, stringent ranges (Havenar-Daughton et al., 2018).

To determine whether anti-major (iGL_311, iGL_317), anti-junctional (iGL_CIS43), and anti-minor (iGL_L9) B cells could be activated by the R21 epitope, we used a recombinant protein encoding the same PfCSP sequence—18 NANP repeats and the C-terminal—as R21, without the HBsAg; this is referred to as “truncated PfCSP (R21-epitope)” below. CD45.2^+^ B cells from each KI line were separately adoptively transferred into congenic CD45.1^+^ WT host mice to achieve a precursor frequency of 5 in 10^5^ B cells, and the recipient mice were then immunized with truncated PfCSP (R21-epitope) and Alhydrogel, and the GC response of splenic B cells was analyzed (Fig. 1 E). The frequency of total GC B cells induced by truncated PfCSP (R21-epitope) was not significantly different across adoptive transfer models (means ranged 0.44–1.0%). Immunization with truncated PfCSP (R21-epitope) selectively activated GC responses from the anti-major repeat iGL_311 and iGL_317 CD45.2^+^ B cells; however, iGL_311 was present at higher frequencies in the GC than iGL_317 (28.2 and 9.1% of GC B cells, respectively). In contrast, truncated PfCSP (R21-epitope) failed to elicit GC responses from either the anti-junctional iGL_CIS43 or anti-minor repeat iGL_L9 B cells (Fig. 1, F and G). To substantiate the in vivo data, we measured the apparent affinity between the different germline Abs and truncated PfCSP (R21-epitope) using biolayer interferometry (BLI). iGL_311 showed the highest affinity to truncated PfCSP (<1.0 × 10^−12^ M), and iGL_317 also displayed measurable affinity (4.07 × 10^−9^ M). In contrast, neither iGL_CIS43 nor iGL_L9 displayed detectable binding (Fig. 1 H).

We then deployed the same approach to assess whether full-length PfCSP, which includes the junctional and minor repeat regions, could activate iGL_CIS43 and iGL_L9 B cells as well as the major repeat iGL_317 and iGL_311 precursors (Fig. 1 I). CD45.2^+^ iGL_317 cells were substantially recruited into the GC, comprising 56.4% of GC B cells. iGL_311 cells were also recruited into the GC, but unexpectedly, recruitment was weaker than that of iGL_317. iGL_CIS43 and iGL_L9 cells were also detected within the GC but at significantly lower frequencies (6.7 and 0.5%, respectively). The GC response from iGL_L9 was ∼100-fold lower than that of iGL_317 and 10-fold lower than iGL_CIS43 (Fig. 1, J and K). We next measured the apparent affinity of germline Abs for full-length PfCSP. Both iGL_311 and iGL_317 bound PfCSP, with iGL_311 showing higher affinity (7.68 × 10^−10^ M and 2.92 × 10^−9^ M). In contrast, iGL_CIS43 and iGL_L9 showed no detectable binding (Fig. 1 L).

Collectively, our results demonstrate that both full-length and truncated PfCSP (R21-epitope) can strongly activate anti-major repeat B cells but offer limited activation of junctional or minor repeat B cells.

### Immunofocusing enhances recruitment and persistence of minor repeat B cells

**Figure S2.**
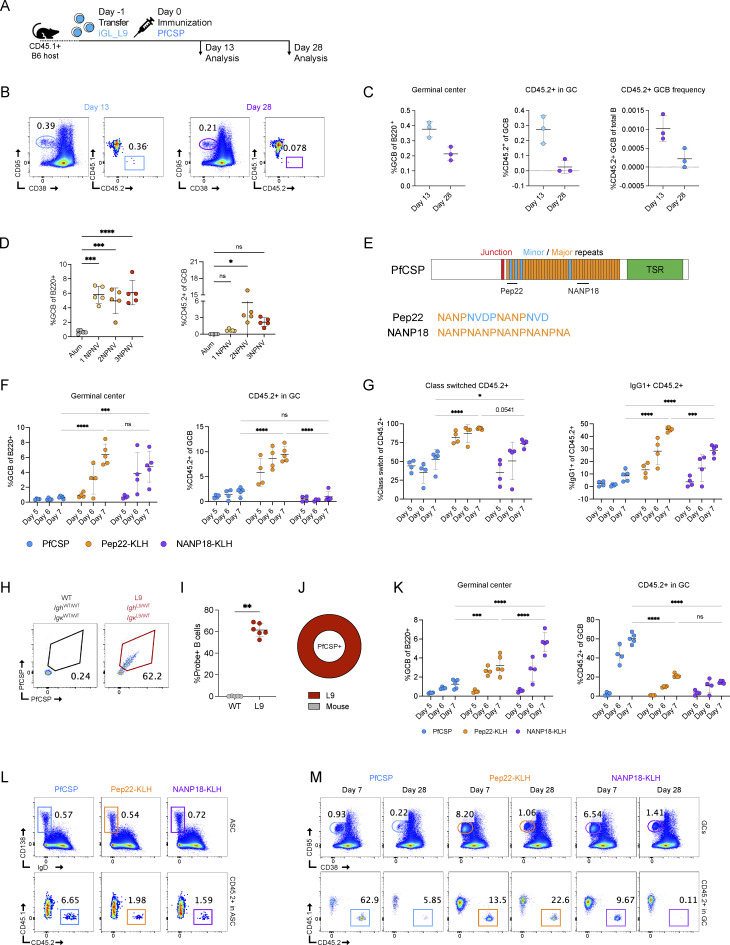
**GC responses of iGL_L9 and L9 B cells to PfCSP and immunofocusing immunogens, related to Fig. 2. (A–C)** iGL_L9 response to PfCSP immunization. **(A)** Schematic of adoptive transfer model to evaluate iGL_L9 responses to PfCSP. **(B)** Representative FACS plot of GC response and CD45.2^+^ B cells in GC. **(C)** Quantification of (left) GC B cells, (center) CD45.2^+^ in GC, and (right) CD45.2^+^ GCB frequency. *N* = 3 mice in each group; one experiment performed. Each symbol represents a different mouse. **(D)** Quantification of (left) GC B cells and (right) CD45.2^+^ in GC as in Fig. 2 B. *N* = 5 mice in each group from one experiment. Each symbol represents a different mouse. One-way ANOVA was applied, and the bars indicate mean ± SD. P > 0.05 no significance (ns), ***P < 0.001, ****P < 0.0001. **(E)** Schematic of PfCSP, minor repeat–focusing Pep22, and major repeat–focusing NANP18. **(F)** Quantification of (left) GC B cells and (right) CD45.2^+^ in GC as in Fig. 2 E. *N* = 4–5 mice in each group from one experiment. Each symbol represents a different mouse. Two-way ANOVA was applied, and the bars indicate mean ± SD. P > 0.05 ns, ***P < 0.001, ****P < 0.0001. **(G)** Quantification of (left) class switched and (right) IgG1^+^ CD45.2^+^ B cells as in Fig. 2 E. *N* = 4–5 mice in each group from one experiment. Each symbol represents a different mouse. Two-way ANOVA was applied, and the bars indicate mean ± SD. P > 0.05 ns, *P < 0.05, ***P < 0.001, ****P < 0.0001. **(H)** Representative PfCSP binding of peripheral blood B cells from high-affinity L9 BCR KI mouse model (*Igh*^*L9/WT*^*, Igκ*^*L9/WT*^). **(I)** Quantification of PfCSP binding of peripheral blood B cells from the L9 mouse model. *N* = 4–6 mice in each group. The data are pooled from two independent experiments. Mann–Whitney’s *t* test was applied, and the bars indicate mean ± SD. **P < 0.01. **(J)** Sequence analysis of sorted total B cells or PfCSP^+^ B cells from the L9 mouse model; red indicates presence of both L9 HC and LC. Total of 26 paired sequences from one mouse. **(K)** Quantification of (left) GC B cells and (right) CD45.2^+^ in GC as in Fig. 2 I. *N* = 4–5 mice in each group from one experiment. Each symbol represents a different mouse. Two-way ANOVA was applied, and the bars indicate mean ± SD. P > 0.05 ns, ***P < 0.001, ****P < 0.0001. **(L)** Representative FACS plot of ASC response and CD45.2^+^ B cells of ASC as in Fig. 2 I. **(M)** Representative FACS plot of GC response and CD45.2^+^ B cells of ASC as in Fig. 2 L.

**Figure 2.**
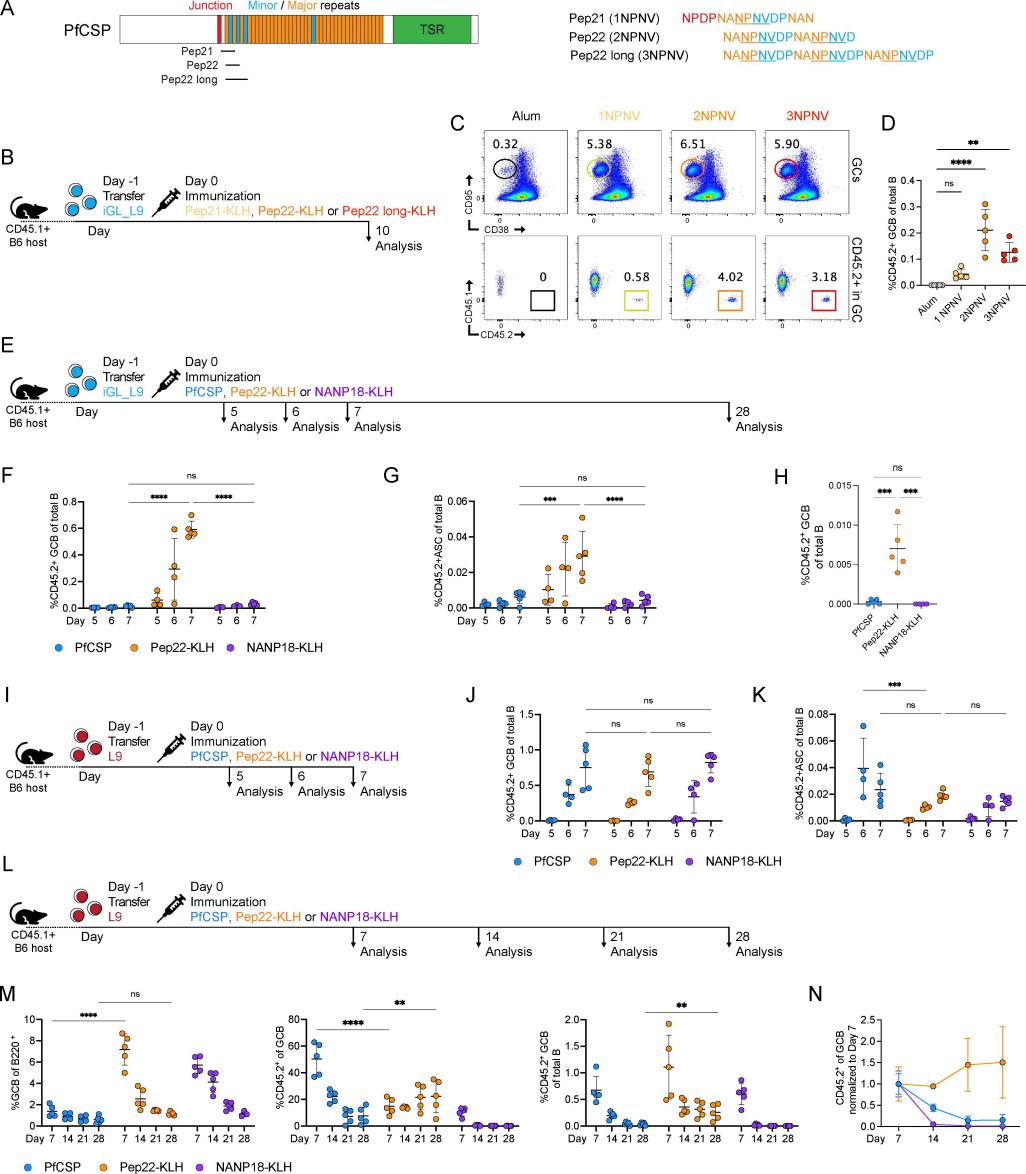
**Response of low-affinity L9 precursor and high-affinity mature L9 B cells to immunization by minor repeat–region immunogens. (A)** Schematic of PfCSP, 1NPNV (Pep21), 2NPNV (Pep22), and 3NPNV (Pep22 long) peptides. **(B)** Schematic of adoptive transfer model to evaluate iGL_L9 responses to 1, 2, or 3 NPNV peptides conjugated with KLH. B cells isolated from iGL_L9 BCR KI mice (CD45.2) were transferred into CD45.1 host mice at a precursor frequency of 5 per 10^5^ cells on day −1, followed by intraperitoneal immunization with Pep21-, Pep22-, or Pep22 long-KLH on day 0. Splenic B cell responses were analyzed on day 10. **(C)** Representative FACS plot of (top) GC response and (bottom) CD45.2^+^ B cells in GC. **(D)** Quantification of CD45.2^+^ GCB frequency. *N* = 5 mice in each group from one experiment. Each symbol represents a different mouse. One-way ANOVA was applied, and the bars indicate mean ± SD. P > 0.05 ns, **P < 0.01, ****P < 0.0001. **(E)** Schematic of adoptive transfer model to evaluate early and late immune response of iGL_L9. B cells isolated from an iGL_L9 BCR KI mouse (CD45.2) were transferred into CD45.1 host mice at a precursor frequency of 5 per 10^5^ cells on day −1, followed by intraperitoneal immunization with full-length PfCSP, Pep22-, or NANP18-KLH on day 0. Early B cell responses were analyzed on day 5, 6, and 7. Late B cell responses were analyzed on day 28. **(F)** Quantification of CD45.2^+ ^GCB frequency on day 5, 6, and 7. *N* = 4–5 mice in each group from one experiment. Each symbol represents a different mouse. Two-way ANOVA was applied, and the bars indicate mean ± SD. P > 0.05 ns, ****P < 0.0001. **(G)** Quantification of CD45.2^+^ ASC frequency on day 5, 6, and 7. *N* = 4–5 mice in each group from one experiment. Each symbol represents a different mouse. Two-way ANOVA was applied, and the bars indicate mean ± SD. P > 0.05 represented as no significance (ns), ***P < 0.001, ****P < 0.0001. **(H)** Quantification of CD45.2^+ ^GCB frequency on day 28. *N* = 4–5 mice in each group from one experiment. Each symbol represents a different mouse. One-way ANOVA was applied, and the bars indicate mean ± SD. P > 0.05 ns, ***P < 0.001. **(I)** Schematic of adoptive transfer model to evaluate early immune response of L9. B cells isolated from L9 BCR KI mouse (CD45.2) were transferred into CD45.1 host mice at a precursor frequency of 5 per 10^5^ cells on day −1, followed by intraperitoneal immunization with full-length PfCSP, Pep22-, or NANP18-KLH on day 0. Early B cell responses were analyzed on day 5, 6, and 7. **(J)** Quantification of CD45.2^+ ^GCB frequency. *N* = 4–5 mice in each group from one experiment. Each symbol represents a different mouse. Two-way ANOVA was applied, and the bars indicate mean ± SD. P > 0.05 ns. **(K)** Quantification of CD45.2^+^ ASC frequency. *N* = 4–5 mice in each group from one experiment. Each symbol represents a different mouse. Two-way ANOVA was applied, and the bars indicate mean ± SD. P > 0.05 ns, ***P < 0.001. **(L)** Schematic of adoptive transfer model to evaluate kinetics of L9 immune response. B cells isolated from L9 BCR KI mouse (CD45.2) were transferred into CD45.1 host mice at a precursor frequency of 5 per 10^5^ cells on day −1, followed by intraperitoneal immunization with full-length PfCSP, Pep22-, or NANP18-KLH on day 0. The kinetics of B cell responses were analyzed on day 7, 14, 21, and 28. **(M)** Quantification of (left) GC B cells, (center) CD45.2^+^ in GC, and (right) CD45.2^+ ^GCB frequency. *N* = 5 mice in each group from one experiment. Each symbol represents a different mouse. Two-way ANOVA was applied, and the bars indicate mean ± SD. P > 0.05 ns, **P < 0.01, ****P < 0.0001. **(N)** Ratio change of CD45.2^+^ in GC normalized to day 7. Bars indicate mean ± SD.

As the mAb L9 is highly protective in humans, we explored further strategies to robustly activate iGL_L9. Full-length PfCSP, although it contains the minor repeat region that L9 preferentially targets, failed to recruit substantial numbers of iGL_L9 B cells into GCs on day 10 in our adoptive transfer model. To rule out the possibility that this reflected the short-time frame of the initial analysis, we extended the analysis to days 13 and 28 (Fig. S2 A) and again found no significant recruitment (Fig. S2, B and C). As ∼96% of full-length PfCSP consists of major repeats, with the L9-targeted minor repeats making up only ∼4% (Fig. 2 A, left) (Zeeshan et al., 2012), we used an immunofocusing approach, similar to our prior studies showing that a junctional peptide (NPDP19) could increase GC responses and affinity maturation in CIS43 precursors compared with full-length PfCSP (Kratochvil et al., 2021). A set of peptides across the junction of PfCSP containing varying numbers of minor repeat epitopes was made (Fig. 2 A, right). As L9 has been observed to bind more strongly to peptides with two NPNV motifs than to those with one (Wang et al., 2020; Tripathi et al., 2023; Martin et al., 2023), we compared KLH-conjugated peptides containing one (Pep21), two (Pep22), or three (“Pep22 long”) NPNV motifs (Fig. 2, A and B). Of note, Pep21 is the junctional epitope that was defined by CIS43, while Pep22 is the minor epitope defined by L9. GC frequencies were similar across groups (∼5–7%), but 1NPNV (Pep21-KLH) recruited significantly fewer CD45.2^+^ iGL_L9 B cells into GCs (0.71%) than 2NPNV (Pep22-KLH, 5.7%) or 3NPNV (Pep22 long-KLH, 2.19%) (Fig. 2 C and Fig. S2 D). This resulted in significantly higher frequencies of CD45.2^+^ GC B cells in the 2NPNV (0.21%) and 3NPNV (0.13%) groups, but not in the 1NPNV group (0.03%) on day 10 (Fig. 2 D).

Based on its comparative advantage in CD45.2 recruitment, Pep22-KLH was selected for comparison against PfCSP and the major repeat peptide NANP18-KLH (Fig. 2 E and Fig. S2 E) to track initiation of the GC response on days 5–7. Pep22-KLH recruited a higher fraction of CD45.2^+^ iGL_L9 B cells into GCs (9.5%) than PfCSP (2.00%; approximately fivefold lower) or NANP18-KLH (0.98%, ∼10-fold lower) on day 7. This resulted in a significantly higher frequency of CD45.2^+^ GC B cells at day 7 in the Pep22-KLH–immunized group (0.59%) compared with PfCSP (0.014%; ∼40-fold lower) or NANP18-KLH (0.032%; ∼18-fold lower) (Fig. 2 F and Fig. S2 F). Pep22-KLH also promoted greater Ab-secreting cell (ASC) differentiation and class switching (Fig. 2 G and Fig. S2 G). Kinetic analysis out to day 28 revealed that iGL_L9 GCB cells persisted only in the Pep22-KLH group (Fig. 2 H).

The findings with low-affinity iGL precursors raised the question of whether Pep22-KLH could sustain GC responses from high-affinity B cells generated through prior malaria exposure. We generated a BCR KI line expressing high-affinity, mature L9, confirmed PfCSP binding (Fig. S2, H and I), and verified HC and LC identity (Fig. S2 J). L9 B cells were adoptively transferred into WT recipients, which were then immunized with PfCSP, Pep22-KLH, or NANP18-KLH (Fig. 2 I). On day 7, GC frequencies were highest in NANP18-KLH (5.67%), intermediate in Pep22-KLH (3.24%), and lowest in PfCSP (1.25%) (Fig. S2 K, left). In contrast, the proportion of CD45.2^+^ L9 GC B cells was highest following immunization with PfCSP (60.5%), followed by Pep22-KLH (21.4%) and NANP18-KLH (14.5%) (Fig. S2 K, right), resulting in a similar overall frequency of CD45.2^+^ GC B cells across all three groups at day 7 (Fig. 2 J). PfCSP induced a significantly stronger early ASC response than either peptide group at day 6 (Fig. 2 K and Fig. S2 L). Longitudinal tracking (Fig. 2 L) showed that GC frequencies fell over time in all groups, but the persistence of mature L9 B cells within GCs differed sharply. NANP18-KLH–recruited cells were almost absent by day 14, and PfCSP-induced cells fell from 50% at day 7–7.7% by day 28. In contrast, Pep22-KLH maintained L9 CD45.2^+^ cells as 14.8% of GC B cells at day 7 and 22.3% by day 28 (Fig. 2 M and Fig. S2 M); Pep22-KLH recipients were thus the only group in which CD45.2^+^ of GCB cells increased relative to day 7 (Fig. 2 N). Thus, focusing the immune response on the minor repeats produced both strong initial recruitment and sustained GC persistence of low-affinity iGL_L9 B cells, as well as long-term GC retention of high-affinity L9 B cells.

### Immunofocusing induces affinity maturation and increased breadth in minor repeat–targeted B cells

**Figure 3.**
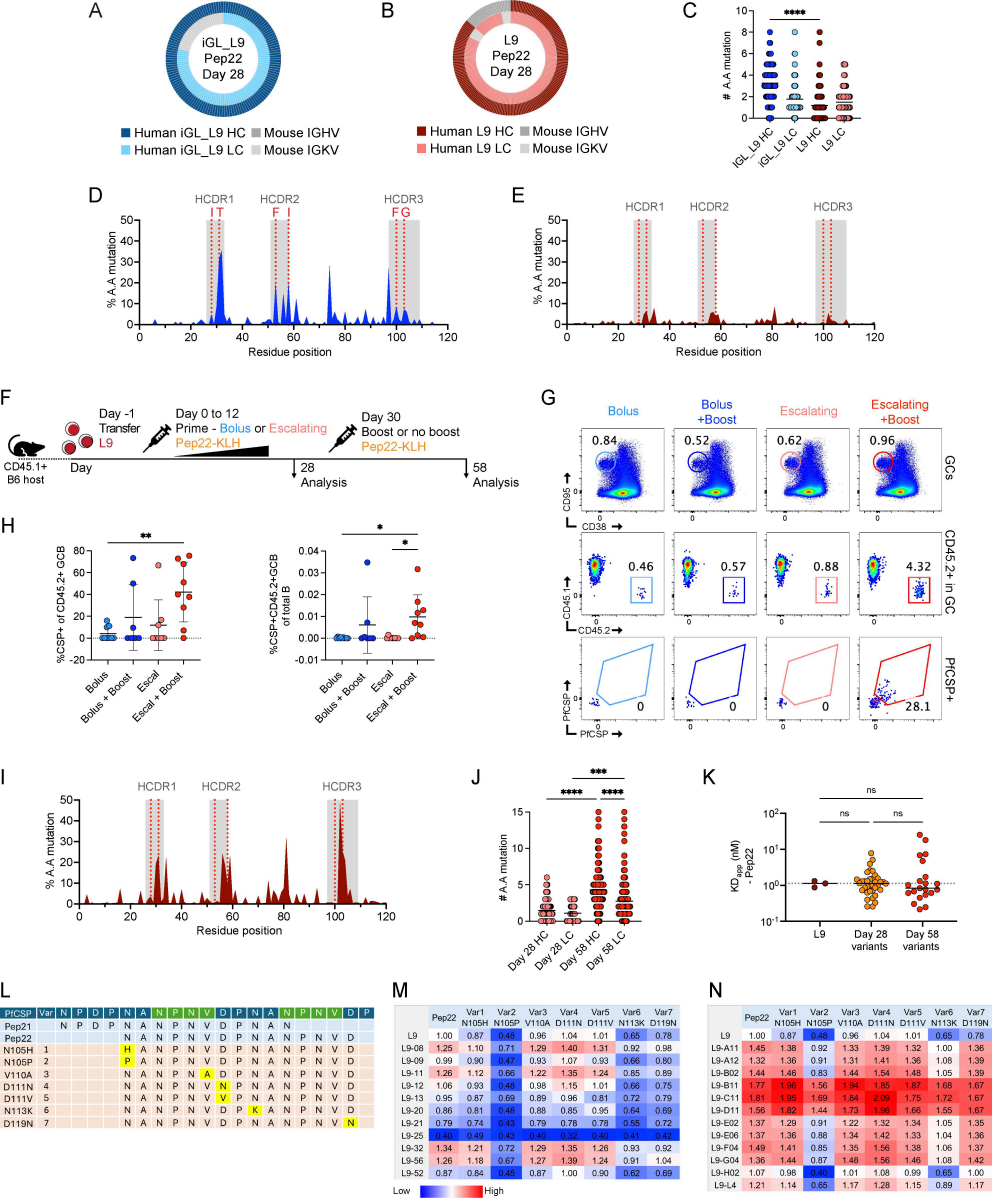
**Mutation and affinity analysis of L9 variants. (A)** Pie chart of HC and LC sequences of sorted CD45.2^+^ GCB cells from Pep22-KLH–immunized iGL_L9 transfer model on day 28, as in Fig. 2 E. Total of 139 paired sequences isolated from four mice. **(B)** Pie chart of HC and LC sequences of sorted CD45.2^+^ GCB cells from Pep22-KLH–immunized L9 transfer model on day 28, as in Fig. 2 I. Total of 218 paired sequences were isolated from five mice. **(C)** Number of mutations in sorted iGL_L9 and L9 B cells. One-way ANOVA was applied, and the bars indicate mean ± SD. ****P < 0.0001. Each dot represents a single HC or LC. **(D)** AA mutation frequencies of HCs from sorted iGL_L9 B cells. Gray area is the complementarity-determining region (CDR), and white area is the framework region (FR). Positions of on-track mutations in CDRs were marked as red dashed lines. Total of 139 paired sequences isolated from four mice, excluding murine sequences, as in Fig. 3 A. **(E)** AA mutation frequencies of HCs from sorted L9 B cells. Gray area is CDR, and white area is FR. Positions of on-track mutations in CDRs were marked as red dashed lines. Total of 218 paired sequences were isolated from five mice, excluding murine sequences, as in Fig. 3 B. **(F)** Schematic of adoptive transfer model to induce extended GC response of L9 B cells by priming with escalating dose and boosting. B cells isolated from L9 BCR KI mice (CD45.2) were transferred into CD45.1 host mice at a precursor frequency of 5 per 10^5^ cells on day −1, followed by intraperitoneal immunization with Pep22-KLH on day 0 as a bolus dose, or with escalating doses of Pep22-KLH administered every other day from day 0 to day 12. The total immunogen dose was identical between the bolus and escalating groups. Mice were then homologously boosted with a bolus dose on day 30. Splenic B cell responses were analyzed on day 58. **(G)** Representative FACS plot showing (top) GC response, (center) CD45.2^+^ B cells in GC, and (bottom) PfCSP^+^ among CD45.2^+^ GCB cells on day 58. **(H)** Quantification of (left) PfCSP^+^ among CD45.2^+^ GCB cells and (right) PfCSP^+^ CD45.2^+^ GCB among total B cells. *N* = 7–9 mice in each group. The data are pooled from two independent experiments. Each symbol represents a different mouse. One-way ANOVA was applied, and the bars indicate mean ± SD. P > 0.05 ns, *P < 0.05, **P < 0.01. **(I)** Mutation frequency of HCs from sorted L9 B cells from escalating + boost immunization group on day 58. Gray area is CDR, and white area is FR. Positions of on-track mutations in CDRs were marked as red dashed lines. Total of 292 paired BCR sequences from three mice. **(J)** Number of mutations of sorted L9 B cells from the escalating immunization group on day 28 or the escalating + boost immunization group on day 58. For day 28, 78 sequences from two mice; for day 58, 292 sequences from three mice. One-way ANOVA was applied, and the bars indicate mean ± SD. ***P < 0.001, ****P < 0.0001. Each dot represents a single HC or LC. **(K)** Comparison of apparent affinity of L9 variant IgGs from day 28 after escalating or day 58 after escalating + boost study to Pep22 by BLI. 36 mAbs from the day 28 study and 21 mAbs from the day 58 study were analyzed. Each dot represents a different mAb. One-way ANOVA was applied, and the bars indicate mean ± SD. P > 0.05 ns. **(L)** Sequences of reported PfCSP variants, which have a mutation in the Pep22 region (MalariaGEN et al., 2023; Zeeshan et al., 2012). **(M)** Pep22 variant probe binding intensity of L9 variant IgGs from day 28 after bolus immunization, as in Fig. 2 I, immobilized on protein A–coated beads. Binding intensity is calculated by mean fluorescence intensity (MFI) to the probes. **(N)** Pep22 variant probe binding intensity of L9 variant IgGs from day 58 post-escalating dose and boost immunization, as in Fig. 3 F, immobilized on protein A–coated beads. Binding intensity is calculated by MFI to the probes.

**Figure S3.**
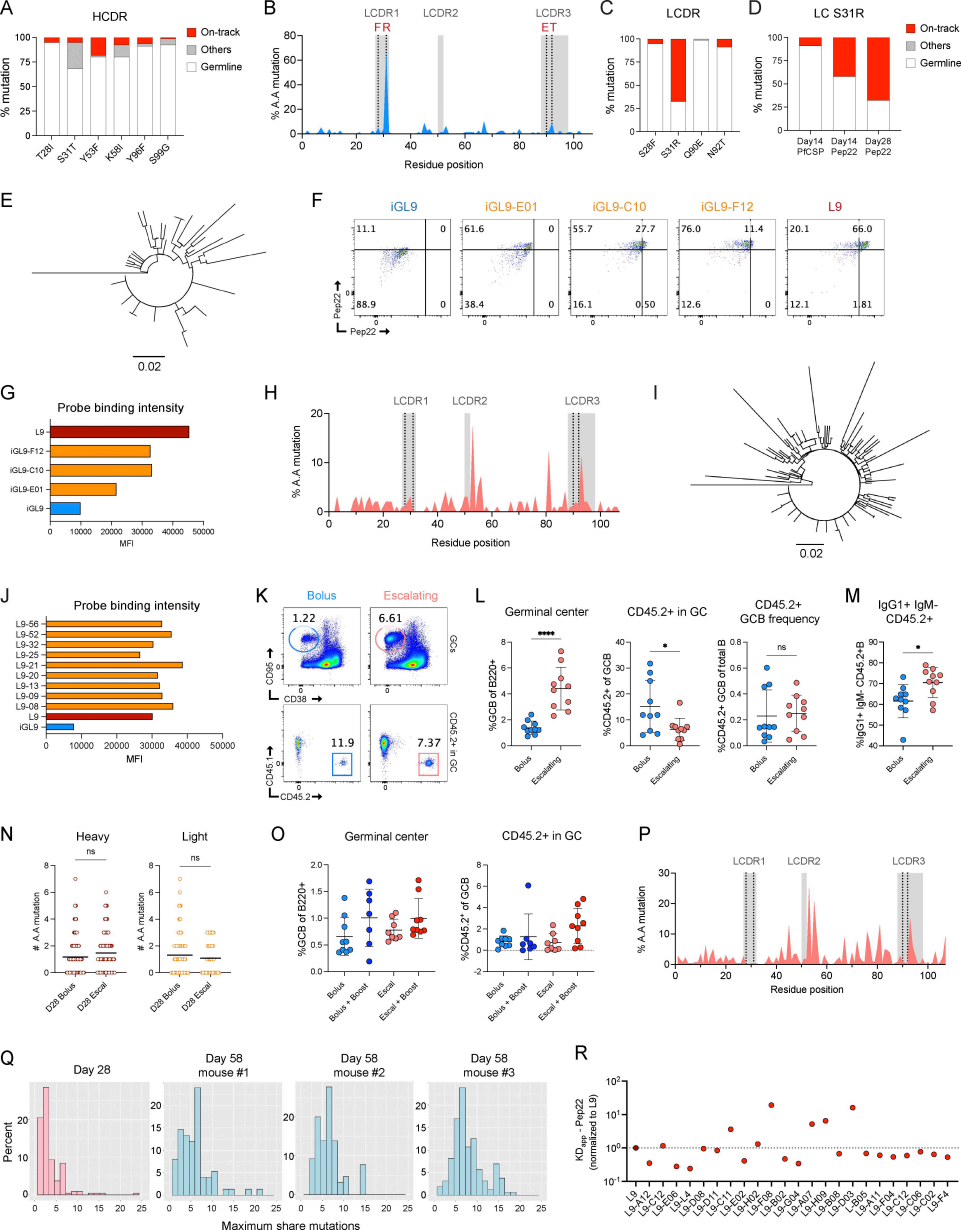
**Mutation and affinity analysis of iGL_L9 and L9 variants, related to Fig. 3. (A)** On-track mutation analysis of HCs from sorted iGL_L9 B cells from Pep22-KLH group at day 28 as in Fig. 2 E. Total of 139 paired sequences pooled from three mice. **(B)** Mutation frequency of LCs from sorted iGL_L9 B cells. Gray area is complementarity-determining region (CDR), and white area is the framework region (FR). Positions of on-track mutations in CDRs were marked as red dashed lines. Total of 139 paired sequences isolated from four mice, excluding murine sequences as in Fig. 3 A. **(C)** On-track mutation analysis of LCs from sorted iGL_L9 B cells. **(D)** Comparison of on-track mutation frequency in LC L31 region after either PfCSP or Pep22 immunization. **(E)** Clonal-lineage tree analysis of iGL_L9 variants at 28 days after immunization. Branch length is representative of AA sequence distance. **(F)** Representative FACS plot of Pep22 probe binding by iGL_L9 variant IgGs. **(G)** Quantification of Pep22 probe binding intensity by iGL_L9 variant IgGs. **(H)** Mutation frequency of LCs from sorted L9 B cells. Gray area is CDR, and white area is FR. Positions of on-track mutations in CDRs were marked as red dashed lines. Total of 218 paired sequences isolated from five mice, excluding murine sequences as in Fig. 3 B. **(I)** Clonal-lineage tree analysis of L9 variants at 28 days after immunization. Branch length is representative of AA sequence distance. **(J)** Quantification of Pep22 probe binding intensity by L9 variant IgGs. **(K)** Representative FACS plot showing GC response and CD45.2^+^ B cells in GC on day 28 as in Fig. 3 F. **(L)** Quantification of (left) GC B cells, (center) CD45.2^+^ in GC, and (right) CD45.2^+^ GCB frequency. *N* = 10 mice in each group. The data are pooled from two independent experiments. Each symbol represents a different mouse. Mann–Whitney’s *t* test was applied, and the bars indicate mean ± SD. P > 0.05 ns, *P < 0.05, ****P < 0.0001. **(M)** Quantification of class-switched IgG1^+^ IgM^−^ CD45.2^+^ B cells. *N* = 10 mice in each group. The data are pooled from two independent experiments. Each symbol represents a different mouse. Mann–Whitney’s *t* test was applied, and the bars indicate mean ± SD. P > 0.05 ns, *P < 0.05. **(N)** Number of mutations of sorted L9 B cell (left) HCs and (right) LCs from bolus or escalating immunization group on day 28. For day 28 bolus, 139 paired sequences were isolated from four mice; for day 28 escalating, 78 sequences from two mice. One-way ANOVA was applied, and the bars indicate mean ± SD. Each dot represents a single HC or LC. **(O)** Quantification of (left) GC B cells and (right) CD45.2^+^ in GC as in Fig. 3 F. *N* = 7–9 mice in each group. The data are pooled from two independent experiments. Each symbol represents a different mouse. One-way ANOVA was applied, and the bars indicate mean ± SD. P > 0.05 ns. **(P)** Mutation frequency of LCs from sorted L9 B cells from escalating + boost immunization group on day 58 as in Fig. 3 F. Gray area is CDR, and white area is FR. Positions of on-track mutations in CDRs were marked as red dashed lines. Positions of on-track mutations in CDRs were marked as red dashed lines. Total of 292 paired BCR sequences from three mice. **(Q)** Number of shared mutations of each BCR sequence within individual mice for the day 28 bolus immunization study and day 58 escalating + boost study. **(R)** Comparison of apparent affinity of L9 variant IgGs from day 58 study to Pep22 by BLI.

To confirm whether mutations associated with malaria neutralization by L9 (Wang et al., 2020) were induced, we sorted CD45.2^+^PfCSP (probe)^+^ GC B cells on day 28 after Pep22-KLH immunization to perform single-cell BCR sequencing. BCRs primarily contained paired iGL_L9 HC and LC sequences (78%, Fig. 3 A) or paired L9 HC and LC sequences (86%, Fig. 3 B), indicating that Pep22 activated target B cells. The HC mutation rate of the low-affinity iGL_L9 was significantly higher than that of high-affinity L9 (Fig. 3 C), suggesting greater potential for affinity maturation in iGL_L9. We compared post-Pep22-KLH immunization HC and LC mutations from PfCSP^+^iGL_L9 B cells with those in mature L9 to evaluate the induction of “on-track” mutations to residues in their mature equivalents. The HC of mature L9 carries six on-track mutations: T28I and S31T in HCDR1, Y53F and K58I in HCDR2, and Y96F and S99G in HCDR3 (Tripathi et al., 2023; Martin et al., 2023), and we detected high mutation frequencies (4.9–31.7%) at these positions (Fig. 3 D). HCDR1 is critical for the homotypic interaction characteristic of L9, and the key on-track mutations T28I (4.9%) and S31T (4.9%) were observed. The HCDR2 region contributes to antigen binding through W52 of *VH3-33* and *VH3-30*, and nearby on-track mutations Y53F (18.3%) and K58I (7.3%) also emerged. The HCDR3 region plays a role in both antigen binding and homotypic interaction, and Y96F (6.1%) and S99G (1.2%) were detected (Fig. S3 A). The LC of mature L9 contains four on-track mutations: S28F and S31R in LCDR1, and Q90E and N92T in LCDR3 (Fig. S3 B). LCDR1 is essential for both antigen binding and homotypic interaction, with the on-track mutations S28F (5%) and S31R (67.6%) observed. In LCDR3, one of the two on-track mutations, N92T (8.8%), was detected (Fig. S3 C). The S31R mutation, the most critical for both antigen binding and homotypic interaction, was strongly induced by Pep22-KLH immunization (42% at day 14 vs. 9% from PfCSP) and became even more prevalent by day 28 (67.6%) (Fig. S3 D). Furthermore, phylogenetic analysis of iGL_L9 variants revealed significant diversification upon Pep22 immunization (Fig. S3 E). To assess whether the mutations induced by Pep22 immunization contributed to affinity maturation, we selected three iGL_L9 variants carrying on-track mutations and measured their approximate affinities using bead assays. Beads coated with each of the three variants exhibited stronger binding to the Pep22 probe compared with beads coated with iGL_L9 (Fig. S3, F and G), indicating that Pep22-KLH induced affinity maturation of the iGL_L9 B cells.

In contrast, despite the long and stable GC response in L9-transferred mice, the overall mutation frequency was very low, and conserved mutations were rarely observed in either HC or LC (Fig. 3, C and E; and Fig. S3 H). Although iGL_L9 AA mutation rates exceeded 20% at seven positions, the highest per-site rate in L9 reached only 17%, and most positions were <10%. L9 nonetheless diversified following Pep22-KLH immunization (Fig. S3 I). To assess whether this diversification translated into affinity maturation, we tested a subset of L9 variants using a bead assay. L9-25 showed reduced affinity, while most variants bound the Pep22 probe with comparable strength with the original L9 (Fig. S3 J). Only a few variants showed improved affinity, and these gains were modest relative to those observed in iGL_L9. Thus, while iGL_L9 variants consistently underwent affinity maturation, the L9 lineage showed a heterogeneous outcome: many variants maintained their original affinity, some lost binding strength, and only a small subset gained affinity—and these gains were smaller than those observed for iGL_L9.

To determine whether longer GC residency might allow for further maturation of high-affinity B cells, we sought to induce a longer GC response by an escalating dose immunization over seven injections from day 0 to day 12 (Tam et al., 2016) (Fig. 3 F). On day 28, the size of the GC was much larger in the escalating dose group (4.4%) than in the group that received a single priming immunization in bolus form (1.4%), likely due to the continuous exposure to antigen. However, the GC CD45.2^+^ fraction was lower in the escalated group (6.4%) than in the bolus group (15.2%), and as a result, there was no significant difference in the frequency of PfCSP^+^ CD45.2 GC B cells between the two groups (Fig. S3, K and L). However, class-switched IgG1^+^ cells had a modestly higher percentage of CD45.2 B cells in the escalating dose group (70.4%) than in the bolus group (61.5%) (Fig. S3 M). PfCSP^+^ CD45.2^+^ GC B cells isolated on day 28 showed no significant difference in mutation frequency between bolus and escalating dose groups (Fig. S3 N). Thus, escalating doses enhanced class-switching but not PfCSP^+^ CD45.2^+^ GC B cell residence or mutation rates.

To ensure antigen availability for GC maintenance, we administered a homologous bolus boost to both groups on day 30 and analyzed the GC response on day 58. GC B cells and CD45.2^+^ ratio in GCs did not differ significantly between post-boost groups (Fig. 3 G and Fig. S3 O). In contrast, the escalating dose + boost group exhibited a significantly higher PfCSP^+^ ratio among CD45.2^+^ GC B cells (42.3%) compared with the bolus + boost group (19%), resulting in the highest frequency of PfCSP^+^ CD45.2^+^ GC B cells among all groups (Fig. 3, G and H). When we isolated PfCSP^+^ CD45.2 GC B cells on day 58 from escalating dose + boost group and examined HC sequences, the mutation frequency was significantly higher on day 58 (HC: 4.29, LC: 2.77 AA mutations) than on day 28 (HC: 1.47, LC: 1.08 AA mutations) from the escalating immunization (Fig. 3, I and J; and Fig. S3 P). Conserved mutations and subclonal expansion were also much more prevalent (Fig. S3 Q). To determine whether the mutations affected affinity, we selected BCR sequences with subclonal expansion, expressed the Abs, and measured their apparent affinity to Pep22. In contrast to day 28 L9 variants, 70% (16 of 23) of the day 58 L9 variants displayed higher affinities to Pep22 than the original L9 (Fig. 3 K and Fig. S3 R).

The finding that some L9 variants gained or lost affinity, together with their underlying sequence diversification, suggested that the repertoire might display broadened reactivity to a wider range of epitopes. We therefore evaluated whether the diversified L9 Abs had acquired affinity for PfCSP variants differing from the 3D7 reference genome (Gardner et al., 2002). To do so, we designed a set of Pep22 variants (Zeeshan et al., 2012) with a range of mutations (Fig. 3 L) and compared the affinity of original L9 against post-bolus day 28 (Fig. 3 M) and escalating + boost day 58 GC B cell variants (Fig. 3 N) using a bead assay. The original L9 displayed similar affinities to Pep22 and three variants with mutations at the V110 or D111 positions (Pep22_Var3, Var4, and Var5) but had lower affinity to other Pep22 variants (Fig. 3 M). L9-08, L9-11, L9-32, and L9-56 displayed slightly higher affinity to Pep22 and all its variants relative to L9. Most L9 variant Abs from day 28 showed similar affinity trends to the original L9, with none displaying significantly superior affinities, though L9-25 exhibited significantly lower affinity (Fig. 3 M). A set of day 58 L9 variants, selected on the basis of higher affinity readings from BLI, were further examined. Most of these also showed higher affinity in the bead assay, and three L9 variants, including L9-B11, C11, and D11, exhibited higher affinity to Pep22 Var2, Var6, and Var7 than did L9 (Fig. 3 N). Thus, high-affinity B cells underwent further affinity maturation when the GC response was extended by boosting, and that prolonged GC reaction enhanced breadth as well as affinity.

### Informatics analyses reveal links between sequences and affinity associated with malaria protection

**Figure 4.**
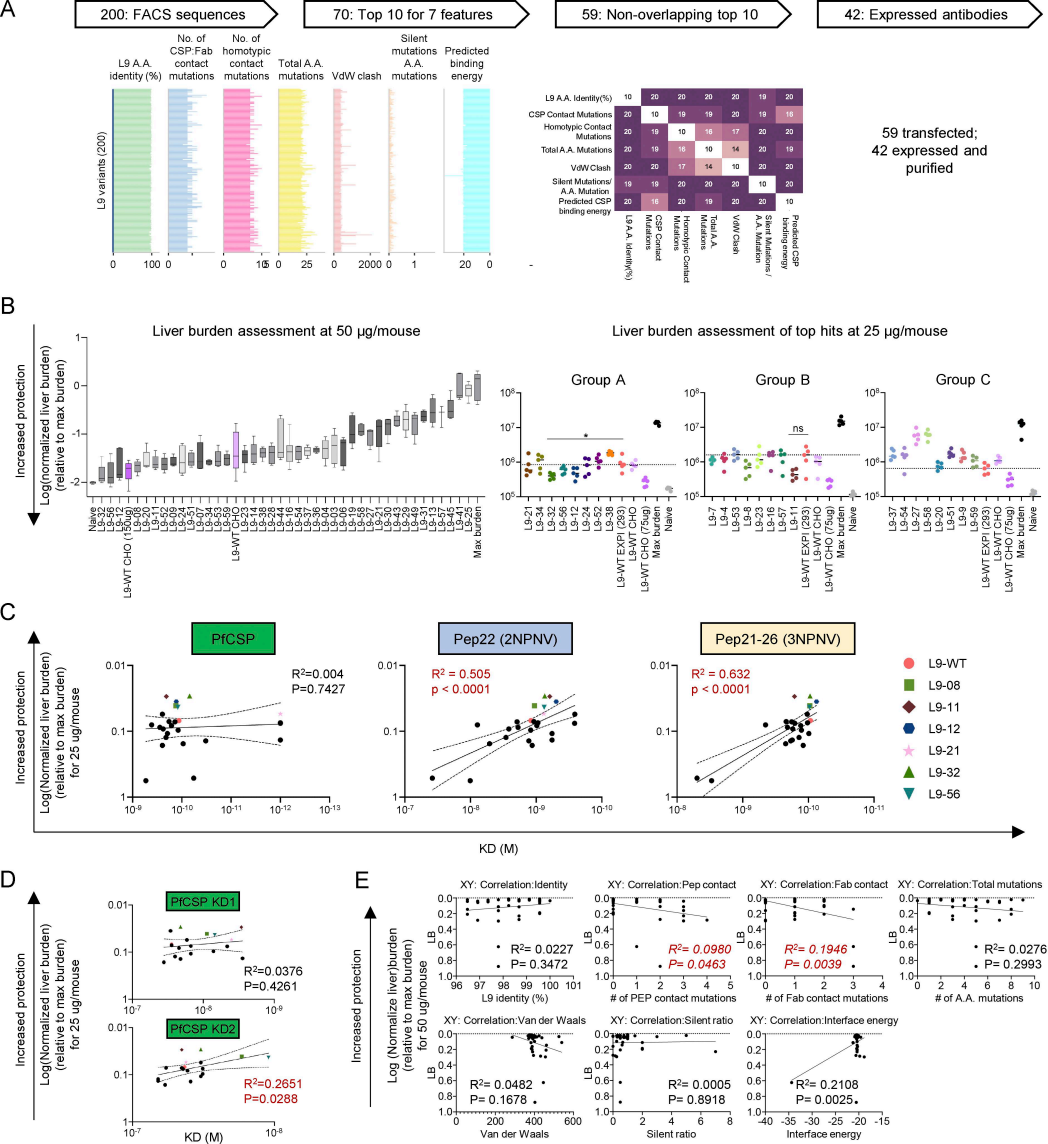
**Informatics-based analyses identify affinity- and sequence-based correlates of improved protection. (A)** Sequence-based sieving of genetic features of 200 mature L9 B cells identified the top 10 sequences for seven features, comprising 59 unique Ab sequences of which 42 were expressed. **(B)** Mice were passively infused with either 50 µg Ab (left; *N* = 5 per group, pooled data from five independent experiments in Fig. S4 A) or 25 µg Ab (right; each dot indicates one mouse) before being challenged with transgenic *P. berghei* SPZ expressing PfCSP and a GFP/luciferase fusion protein. Bioluminescent quantification of liver burden is shown 42 h after challenge, with each group of Abs assessed with mature L9 control expressed in EXPI and CHO cells, respectively. The error bars in the box plot represent minimum and maximum. P > 0.05 represents no significance (ns), *P < 0.05 as calculated by a two-tailed Mann–Whitney test assuming non-normal distribution. The dotted line in the right panel represents the median flux of L9 control. **(C)** BLI affinity for 42 expressed Abs was measured against PfCSP, minor repeat peptides containing two or three NPNV repeats, respectively, and correlated to normalized liver burden (y axis), as assessed at 25 µg/ml. Solid line represents the simple linear regression fit; dotted lines indicate the 95% confidence interval. R^2^ and two-sided P values were calculated by simple linear regression. **(D)** ITC affinities for selected Abs were measured against PfCSP and correlated to normalized liver burden (y axis), as assessed at 25 µg/ml (see Fig. S4 C). Solid line represents the simple linear regression fit; dotted lines indicate the 95% confidence interval. R^2^ and two-sided P values were calculated by simple linear regression. **(E)** Correlations between seven genetic features chosen for sequence sieving and normalized protection, as assessed at 50 µg/ml. The correlation between the normalized liver burden and various kinetics, sequence, and structural properties was calculated using two-tailed Pearson’s correlation method.

**Figure S4.**
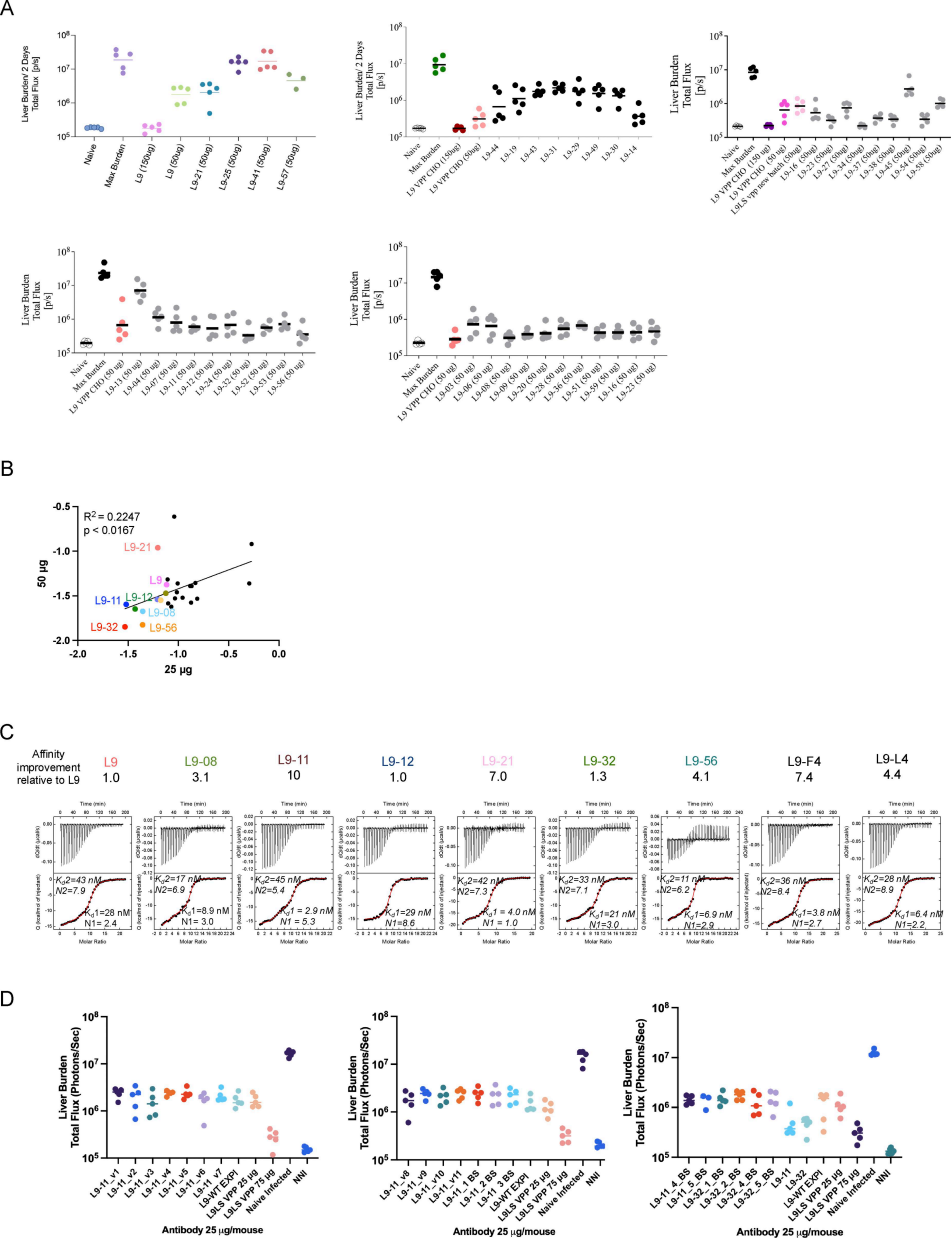
**Additional in vivo efficacy and biophysical characterization of L9-derived Abs, related to Figs. 4, 5 and 6. (A)** Mice were passively infused with 50 µg Ab before being challenged with transgenic *P. berghei* SPZ expressing PfCSP and a GFP/luciferase fusion protein. Bioluminescent quantification was performed for each mouse on day 2 after challenge. *N* = 5 per group. Each experiment was performed independently. Bar represents median flux. **(B)** Correlation between protective efficacy assessed at 50 and 25 µg doses performed using simple linear regression. **(C)** Isothermal calorimetry titrations of PfCSP with various L9-derived Abs at pH 7.4 and 25°C. The affinities and stoichiometry are shown for both K_D_1 and K_D_2. **(D)** Mice were passively infused with 25 µg Ab before being challenged with transgenic *P. berghei* SPZ expressing PfCSP and a GFP/luciferase fusion protein. Bioluminescent quantification of liver burden is shown 42 h after challenge, with each group of Abs assessed with mature L9 control expressed in EXPI and CHO cells, respectively. *N* = 5 mice in each group. Each experiment was performed independently.

As the elicitation of further affinity maturation would likely be more challenging with a higher affinity starting point, to delve deeper into whether Pep22 immunization enhanced the quality of Ab responses, we performed an informatics analysis of 200 Ab sequences isolated from the post-bolus day 28 mature L9 transfer group. This analysis aimed to identify top sequence variants by assessing seven potential features that might correlate with protection, including (1) % identity to the mature L9 Ab, (2) the number of Ab contacts with PfCSP based on modeling of the L9-PfCSP structure (Tripathi et al., 2023), (3) the number of Ab homotypic contacts also based on modeling, (4) the number of AA mutations, (5) the number of modeled van der Waals clashes, (6) the ratio of silent versus AA mutations, and (7) the predicted binding energy (Fig. 4 A). The sieving process identified 59 variant L9-Ab sequences as the top sequence variants, of which 42 could be expressed and purified (Fig. 4 A). We assessed the effectiveness of these 42 Abs in reducing liver burden following an intravenous challenge with transgenic *Plasmodium berghei* SPZ expressing PfCSP and green GFP-luciferase (GFP-Luc) (Flores-Garcia et al., 2019). Based on the quantitatively assessed reduction in liver burden by the different Ab variants, we selected those that showed the greatest reduction for in-depth structural analysis. This approach allowed us to identify sequence features that correlate with enhanced protective efficacy. Among the 42 Abs, at 50 µg per mouse, ∼14 Abs showed comparable or slightly improved reduction of liver burden relative to the mature WT L9 Ab, indicating highly protective activity (Fig. 4 B, left, and Fig. S4 A). To refine our selection and identify the most promising candidates, we reassessed 24 of these variants at a reduced dose of 25 µg per mouse, separated into three groups (A, B, and C) of eight Abs each, along with the control Ab L9 expressed in EXPI (293) and CHO cells. This secondary screen helped narrow the pool to a subset of Abs, including L9-11 and L9-32, both of which often ranked among those that conferred the greatest reduction in liver burden (Fig. 4 B, right). While protective efficacy was generally correlated between the two doses (Fig. S4 B), distinguishing fine differences among highly potent Abs would require assessment with a substantially higher number of mice to overcome intrinsic variation between animals. Correlations of affinity versus protection revealed that Ab affinity to PfCSP, as measured by BLI, did not correlate significantly; however, higher affinity to peptide 22 (two NPNV repeats) or peptide 21–26 (3 NPNV repeats) did correlate significantly with protection (Fig. 4 C). Moreover, when PfCSP affinities were measured by isothermal titration calorimetry (ITC), multiple binding events were observed, generally governed by at least two dissociation constants (Fig. S4 C). When we assessed correlations with both of these, we observed the affinity associated with the second dissociation constant (KD2) to correlate significantly with protection (Fig. 4 D), indicating an interplay between affinity and protection. Furthermore, in terms of the seven sequence-based features, significant correlations were observed with only two features: mutations on Ab-PfCSP contacts and mutations at homotypic-contact sites, both of which were negatively correlated with improved protection; such negative correlation suggested SHM in these regions to lead to reduced protection (Fig. 4 E), likely because both PfCSP contacts and homotypic contacts have already been substantially optimized by SHM in the mature L9 Ab.

### Cryo-EM analysis visualizes structural basis of protection by L9 variants

**Figure 5.**
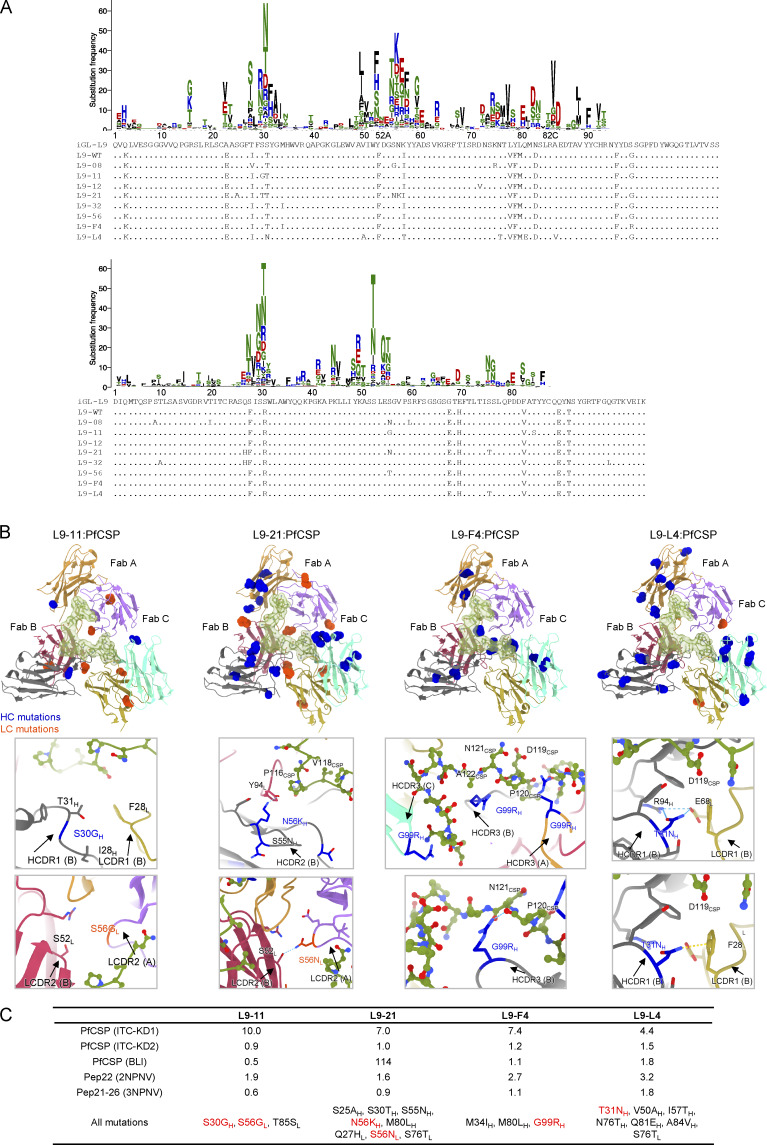
**Structural and sequence features of affinity-matured L9 variants in complex with PfCSP. (A)** Sequence alignment of top L9 variants relative to WT-L9, with corresponding mutational frequency profiles for the HC (top) and LC (bottom). Residue positions are shown along the V-gene, and the height of each letter reflects the frequency of AA substitutions at that position across selected variants. Alignments below indicate specific substitutions in individual variants, highlighting recurrent mutation hotspots in both chains. **(B)** Cryo-EM structures of PfCSP in complex with selected high-affinity L9 variants (L9-11, L9-21, L9-F4, and L9-L4). Three Fab molecules (A–C) engage PfCSP, forming a homotypic interface. HC mutations are highlighted in blue and LC mutations in orange. PfCSP is shown in green; the majority of the protein is disordered, with only a ∼27-residue segment resolved in the density. Insets show close-up views of representative mutations, illustrating their structural context and contributions to binding, including hydrogen bonding, CH-π interactions, and stabilization of the homotypic Fab–Fab interface. **(C)** Binding affinities of top L9 variants measured by ITC and BLI against PfCSP and peptide constructs. Corresponding mutations for each variant are listed below, with key substitutions highlighted in red. Mutations associated with improved affinity map to regions involved in antigen binding or inter-Fab interactions.

After identifying key correlates of protection through informatics screening and affinity analyses, we sought to understand the structural basis of these findings. L9 recognizes a conformational epitope on PfCSP and displays homotypic interactions (Martin et al., 2023; Tripathi et al., 2023), indicating a trimeric binding modality. To examine how specific mutations affect binding and contribute to enhanced protection, we determined cryo-EM structures of antigen-binding fragments (Fabs) of selected L9 variants in complex with PfCSP (Fig. 5 A). In particular, we selected two variants from day 28, L9-11 and L9-21, as L9-11 often trended toward improved protection, and the BLI affinity for L9-21 was two orders of magnitude higher than that of WT L9. We also selected two variants from day 58, L9-F4 and L9-L4, as these had been observed to be particularly expanded on day 58 of the GC in independent mice and showed improved apparent affinity for Pep22 (Fig. S3 R).

**Figure S5.**
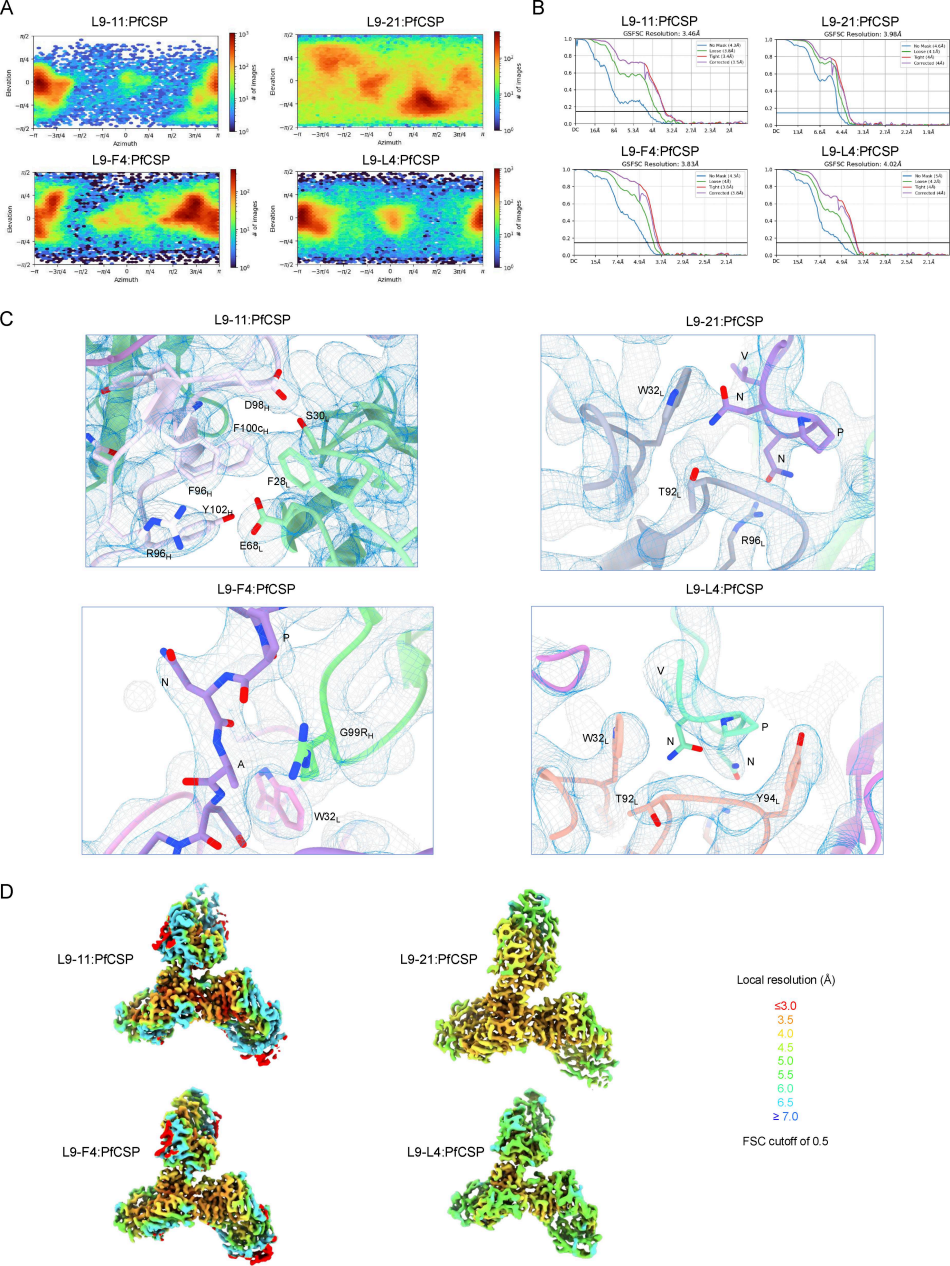
**Cryo-EM characterization of L9-11, L9-21, L9-F4, and L9-L4 in complex with PfCSP, related to Fig. 5 and Table S2. (A)** Particle orientation distributions for the final refinements of the L9-11, L9-21, L9-F4, and L9-L4 complexes, shown as heatmaps. **(B)** Gold-standard Fourier shell correlation (GSFSC) curves for the corresponding reconstructions, yielding overall resolutions of 3.46 Å (L9-11), 3.98 Å (L9-21), 3.83 Å (L9-F4), and 4.02 Å (L9-L4) following nonuniform refinement with C1 symmetry. **(C)** Representative cryo-EM densities highlighting key interactions resolved in each structure: the L9-11 homotypic interface, the L9-21 NPNV–LC interaction, the L9-F4 G99R_H_-PfCSP interaction, and the L9-L4 NPNV–LC interaction. **(D)** Local resolution maps of the final reconstructions for each complex, contoured at 0.35 (5.7σ). Resolution estimation was generated through cryoSPARC using a Fourier Shell Correlation (FSC) cutoff of 0.5.

We determined cryo-EM structures of L9-11, L9-21, L9-F4, and L9-L4 in complex with PfCSP, and pairwise comparisons with the original L9-CSP structure show that the overall backbone is highly conserved (Cα RMSD ∼0.7–0.8 Å), indicating no substantial global rearrangements. The structure of Fab L9-11 with PfCSP by single-particle cryo-EM was determined to a resolution of 3.5 Å (Fig. 5 B, far left panel, and Fig. S5). There were only three mutations of L9-11 relative to WT-L9: S56G_L_, T85S_L_, and S30G_H_ (for clarity, we have added a subscript, H or L, defining whether the mutation is on the HC or LC) (Fig. 5 B, far left panel); all of these mutations reduced the size of the Ab, potentially enabling better induced fit. L9-21 had eight mutations relative to WT-L9 (Q27H_L_, S56N_L_, S76T_L_, S25A_H_, S30T_H_, S55N_H_, N56K_H_, and M80L_H_), and we determined its structure in complex with PfCSP to a resolution of 4.0 Å (Fig. 5 B, second panel from left, and Fig. S5). Three of these mutations were located at the homotypic interface between Fabs (two from HC and one from LC), and two HC mutations were closer to the PfCSP epitope. In particular, the N57K_H_ mutation in CDRH2 interacted with Y94_L_ via a hydrogen bond, and Y94_L_ had a critical CH-π interaction with P116_CSP_ (Fig. 5 B second panel).

The Ab L9-F4 has three mutations (M34I_H_, M80L_H_, and G99R_H_), with G99R_H_ on CDRH3 close to PfCSP. We determined the structure at a resolution of 3.8 Å (Fig. 5 B, third panel from left, and Fig. S5). Structural analysis revealed a side-chain hydrogen bond between G99R_H_ and the backbone carbonyl of PfCSP, explaining the improved affinity relative to WT-L9 (Fig. 5 B, third panel). Ab L9-L4 has seven mutations (S76T_L_, T31N_H_, V50A_H_, I57T_H_, N76T_H_, Q81E_H_, and A84V_H_), with one mutation at the homotypic interface. The structure obtained at a resolution of 4.0 Å (Fig. 5 B, far right panel, and Fig. S5) revealed that T31N_H_ on CDRH1 formed a network of hydrogen bonds with R94_H_ and E68_L_. T31N_H_ also formed a critical NH-π interaction with F28_L_. As both F28_L_ and E68_L_ have been shown to be critical for the stability of the homotypic interface (Tripathi et al., 2023), the introduction of the T31N_H_ mutation likely further enhances the homotypic interface (Fig. 5 B, far right panel).

Overall, the cryo-EM structures of L9-11, L9-21, L9-F4, and L9-L4 in complex with PfCSP visualized critical mutations leading to improved affinity, as measured by ITC and BLI (Fig. 5 C).

### Kinetics-based design yields Abs with further improved affinity

**Figure 6.**
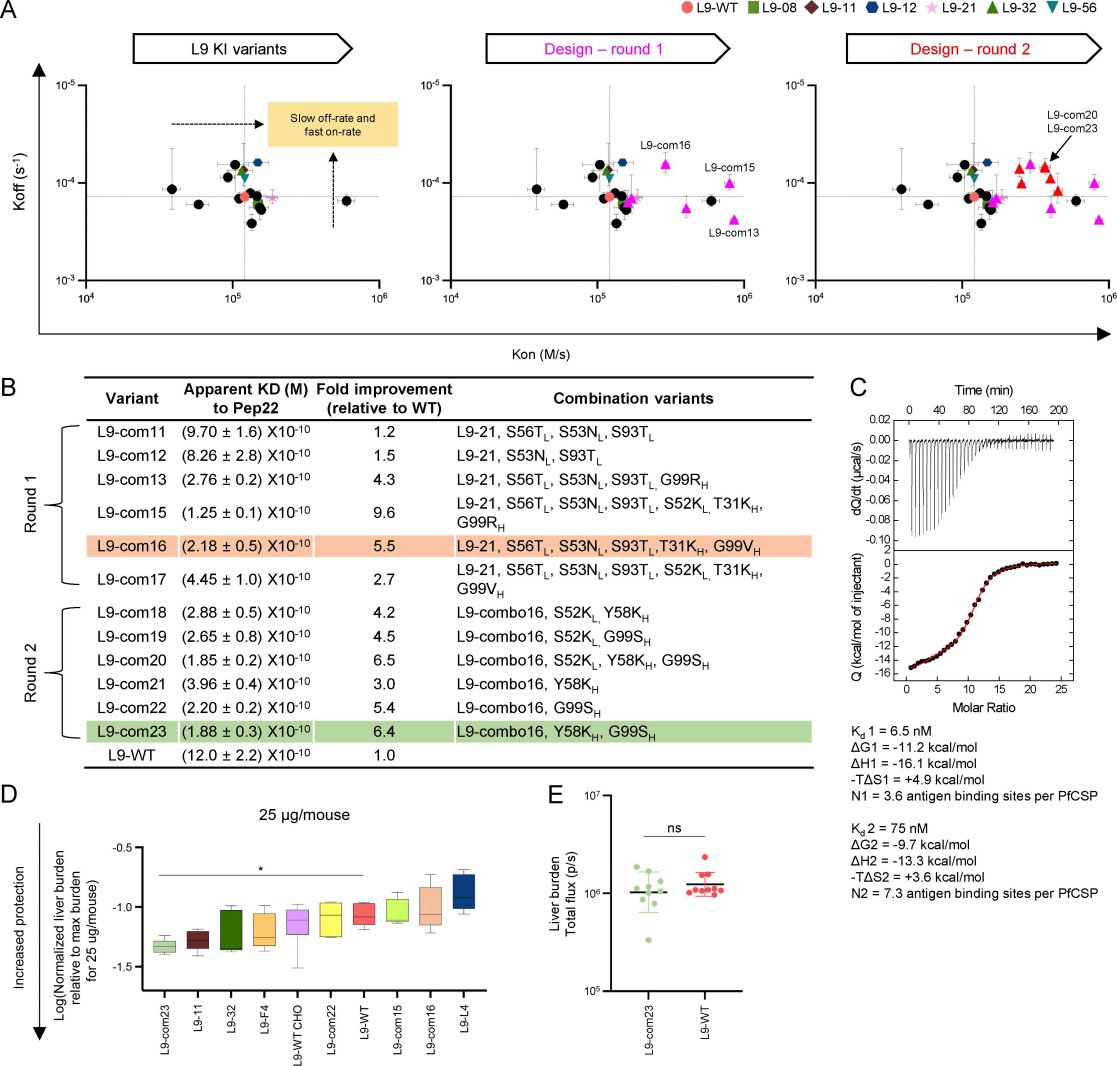
**Kinetics-guided design yields L9 variants with improved affinity and protection. (A)** Binding kinetics (kon vs. koff) of L9 variants measured by BLI. Left, parental variants; middle, first-round designs; right, second-round designs derived from L9-com16. Variants with improved affinity shift toward slower off-rates and/or faster on-rates, with L9-com15/16 identified in round 1 and L9-com23 emerging from round 2. **(B)** Sequences and apparent affinities (KDapp) of designed variants relative to the L9-21 template, measured by BLI against Pep22. Combination variants show up to ∼10-fold improved affinity over WT-L9. Error bars indicate replicate variability. **(C)** ITC binding of L9-com23 to PfCSP. Top, raw thermogram; bottom, fitted isotherm with derived thermodynamic parameters, consistent with high-affinity, multivalent binding. **(D)** Mice were passively infused with 25 µg of one of a panel of 10 Abs before being challenged with transgenic *P. berghei* SPZ expressing PfCSP and a GFP/luciferase fusion protein. Bioluminescent quantification was done for each mouse on day 2 after challenge. *P < 0.05 as calculated by one-way ANOVA test with Dunn’s correction. L9-WT CHO refers to Ab L9 expressed using CHO stable pools, whereas L9-WT refers to Ab L9 expressed in a transient CHO cell line, similar to the mock Abs. **(E)** Repeat liver burden assessment of L9-com 23 with *n* = 10 mice/group from one experiment. This experiment was conducted independently of Fig. 6 D. *P < 0.05 as calculated by one-way ANOVA test with Dunn’s correction.

As the peptide affinity of L9 variant Abs did correlate with improved malaria protection, we tested combinations of changes from each of the separate Abs to see if we could further improve both peptide affinity and malaria protective efficacy. As we observed anticorrelations with mutations at peptide contacts or at homotypic Fab contacts, which suggested that SHM at these positions would lead to reduced protection, we assessed combinations with only day 28 variants. Lastly, when examining the kinetics of peptide recognition, we observed variable on- and off-rates. Notably, no natural variants derived from sequencing of B cells ex vivo exhibited both a faster on-rate and a slower off-rate to Pep22 relative to L9 (Fig. 6 A). We chose to focus on making changes to the three variants from day 28, which consistently showed the highest level of malaria protection (L9-11 and L9-32) or had the fastest on-rate (L9-21).

The initial designs utilized L9-11 and L9-32 as templates, and shared mutations observed within the top 15 Abs were added on top of the template Abs. These combination Abs were expressed (Table S1) and assessed for in vivo efficacy. None of these combinations improved the protective efficacy compared with the template Abs (Fig. S4 D). The second set of combinations based on L9-21 was expressed (Fig. 6 B and Table S1), and the affinity to Pep22 was assessed by BLI. From this set, three combinations with L9-21 yielded 4.3–9.6-fold improved affinity (L9-com13, 15, and 16).

While L9-com15 had the highest Pep22 affinity (9.6-fold improved over L9), this was mostly due to an improved on-rate. L9-com16 had more balanced on- and off-rates (Fig. 6 A, middle panel) and was chosen as the template for the next round of optimization. We added additional mutations to L9-com16, identifying two variants, L9-com20 and L9-com23, both with over sixfold improved Pep22 affinity. The analysis of L9-com23 by ITC revealed it to have substantially high affinity (4.3-fold improved KD1, but a similar KD2) to WT L9 (Fig. 6 C). Of these, L9-com23, when assessed at 25 µg/mouse, showed a significant trend toward improved malaria protection relative to WT L9 (Fig. 6 D). However, when L9 and L9-com23 were independently compared directly, this improvement in liver burden was not significantly better than L9 (Fig. 6 E).

Overall, analysis of Ab kinetics led to the design of L9 variants with significantly improved affinity (∼10-fold for L9-com15 and ∼ 6.4–6.5-fold for L9-com23 and L9-com20). However, these improvements did not translate into significantly enhanced in vivo protection, suggesting that higher affinity alone does not significantly enhance protection, which highlights the difficulty in demonstrating further improvements among already highly protective Abs in this model.

### Immunofocusing peptide provides epitope diversity of anti-CSP B cells

**Figure 7.**
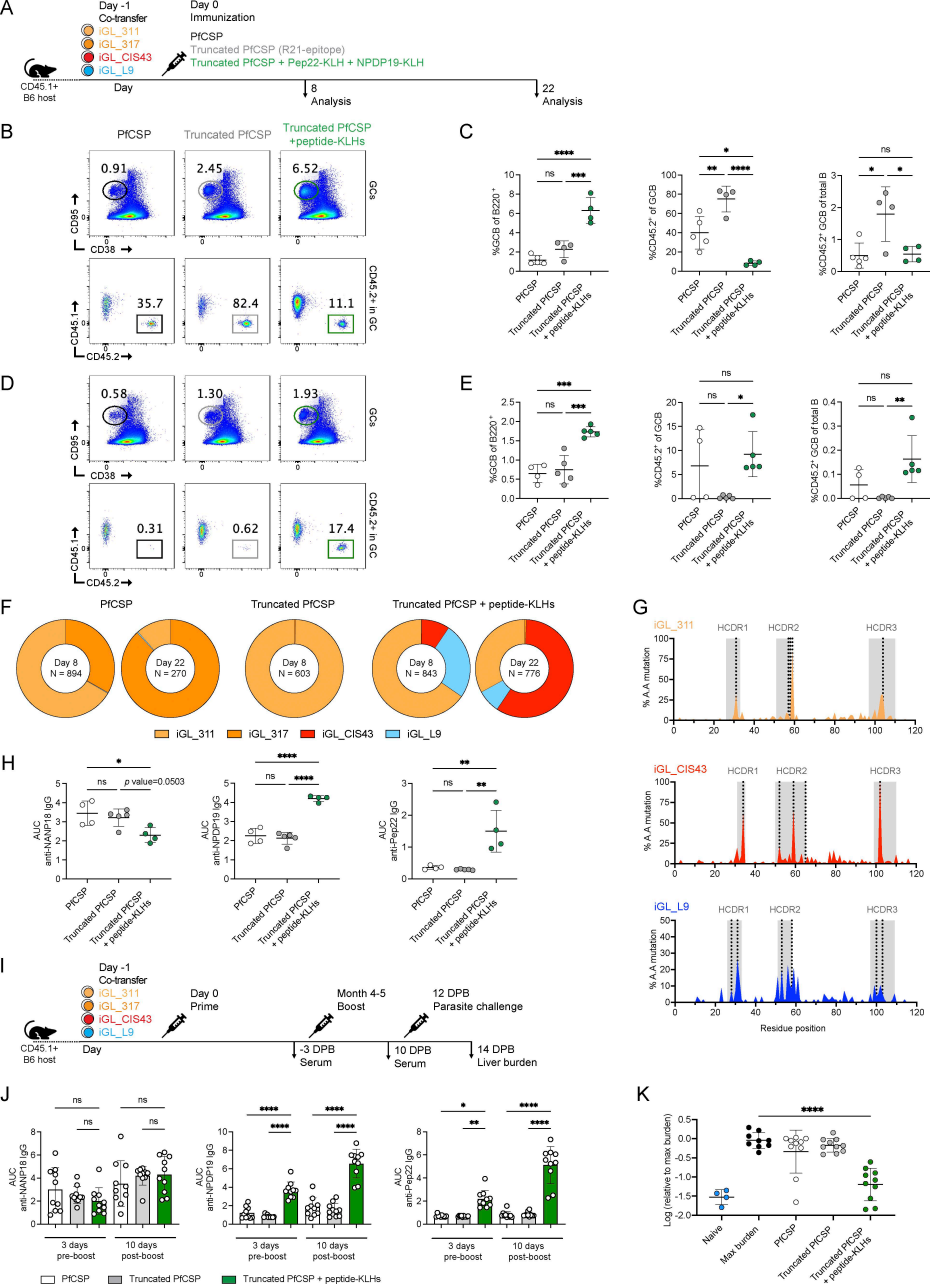
**Balanced immune responses and anti-parasite protection generated through the administration of peptide cocktails. (A)** Schematic of mixed adoptive transfer model to test clonal diversity after immunization. B cells isolated from each BCR KI mouse (CD45.2) were co-transferred into CD45.1 host mice at a precursor frequency of 5 per 10^5^ cells on day −1, followed by intraperitoneal immunization with full-length PfCSP, truncated PfCSP, or truncated PfCSP with Pep22-and NPDP19-KLH on day 0. Splenic B cell responses were analyzed on days 8 and 22. **(B)** Representative FACS plots showing (top) GC (CD95^+^CD38^−^) and (bottom) CD45.2^+^ response in GC on day 8. **(C)** Quantification of (left) GC B cells, (center) CD45.2^+^ in GC, and (right) CD45.2^+^ GCB frequency on day 8. *N* = 4–5 mice per group from one experiment. Each symbol represents a different mouse. One-way ANOVA was applied, and the bars indicate mean ± SD. P > 0.05 represented as no significance (ns), *P < 0.05, **P < 0.01, ***P < 0.001, ****P < 0.0001. **(D)** Representative FACS plots showing (top) GC (CD95^+^CD38^−^) and (bottom) CD45.2^+^ response in GC on day 22. **(E)** Quantification of (left) GC B cells, (center) CD45.2^+^ in GC, and (right) CD45.2^+^ GCB frequency on day 22. *N* = 4–5 mice per group from one experiment. Each symbol represents a different mouse. One-way ANOVA was applied, and the bars indicate mean ± SD. P > 0.05 ns, *P < 0.05, **P < 0.01, ***P < 0.001. **(F)** Pie chart of clones of sorted CD45.2^+^ GCB cells from mixed adoptive transfer model on day 8 and day 22. The sequences were pooled from two to five mice per group. **(G)** AA mutation frequencies of HCs of each clone from CD45.2^+^ GCB cells isolated from the truncated PfCSP (R21-epitope) + peptides group at day 22. Gray area is CDR, and white area is FR. Black dashed lines denote positions of on-track mutations in CDRs. **(H)** ELISA quantification of IgG specific to the (left) major, (center) minor, or (right) junctional epitope peptides from sera in the mixed adoptive transfer model on day 22. *N* = 4–5 mice per group from one experiment. Each symbol represents a different mouse. One-way ANOVA was applied, and the bars indicate mean ± SD. P > 0.05 ns, *P < 0.05, **P < 0.01, ****P < 0.0001. **(I)** Schematic of in vivo parasite challenge in mixed adoptive transfer mouse models to evaluate protection following immunization. B cells isolated from each BCR KI mouse (CD45.2) were co-transferred into a single CD45.1 host mouse at a precursor frequency of 5 per 10^5^ cells on day −1, followed by intraperitoneal immunization with full-length PfCSP, truncated PfCSP, or truncated PfCSP supplemented with Pep22- and NPDP19-KLH on day 0 (prime) and month 4 or 5 (boost). Serum was collected either 3 days pre-boost or 10 days post-boost (DPB). Mice were challenged with 2,000 transgenic *P. berghei* SPZ expressing PfCSP-luciferase at 12 DPB, and protective efficacy was quantified by liver-stage parasite burden at 14 DPB. **(J)** ELISA quantification of IgG specific to the (left) major (anti-NANP18), (center) junctional (anti-NPDP19), or (right) minor (anti-Pep22) epitope peptides from sera in the mixed adoptive transfer models either 3 days pre-boost or 10 days post-boost (DPB). The data are pooled from two independent experiments; total *N* = 9–10 mice in each group. Each symbol represents a different mouse. One-way ANOVA was applied, and the bars indicate mean ± SD. P > 0.05 ns, *P < 0.05, **P < 0.01, ****P < 0.0001. **(K)** Bioluminescent quantification of liver burden is shown 42 h after challenge. *N* = 9–10 mice in each group. The data are pooled from two independent experiments. Each symbol represents a different mouse. One-way ANOVA was applied, and the bars indicate mean ± SD. ****P < 0.0001.

Assessing the responses of diverse anti-CSP B cell precursors within a single host better reflects the complex competitive environment of human physiology. To investigate the diversity of anti-CSP epitope responses following immunization, we adoptively co-transferred into WT mice 4 distinct B cell precursors specific for different PfCSP regions—two anti-major repeat clones (iGL_311 and iGL_317), an anti-junctional clone (iGL_CIS43), and an anti-minor repeat clone (iGL_L9), thereby generating a mini-repertoire of human antimalaria precursors. Recipients were then immunized with either (1) full-length PfCSP, (2) the truncated PfCSP (R21-epitope) defined above, or (3) a combination of truncated PfCSP (R21-epitope) with junctional region (NPDP19-KLH) and minor repeat region (Pep22-KLH) peptide immunogens, using Alhydrogel as the adjuvant (Fig. 7 A).

At day 8, GC frequencies were higher in the truncated PfCSP + peptides group (6.3%) compared with truncated PfCSP (2.3%) alone or full-length PfCSP (1.2%). Recruitment of CD45.2^+^ B cells into the GC was highest in the truncated PfCSP group (75%), intermediate in the full-length PfCSP group (40%), and lowest in the truncated PfCSP (R21-epitope) + peptides group (8.4%). As a result, the truncated PfCSP (R21-epitope) group showed a threefold higher frequency of CD45.2^+^ GC B cells (1.8%) compared with the full-length PfCSP (0.5%) or truncated PfCSP + peptides group (0.55%) (Fig. 7, B and C). Thus, truncated PfCSP, with or without the targeted junctional and minor repeat peptides, induced a stronger early GC response than full-length PfCSP from transferred B cells encompassing the major, minor, and junctional iGLs. At day 22, GC B cells remained highest in the truncated PfCSP + peptides group (1.7%), followed by truncated PfCSP (0.75%) and full-length PfCSP (0.65%). However, CD45.2^+^ GC B cells in the truncated PfCSP group were close to undetectable, and only two out of four mice in the full-length PfCSP group had CD45.2^+^ GC B cells. In contrast, the truncated PfCSP + peptides group maintained a markedly higher GC proportion (9.23%) and exhibited the highest frequency of total CD45.2^+^ GC B cells among all groups (0.16%) (Fig. 7, D and E).

To determine which clones were recruited into the GC following immunization, we analyzed GC composition by single-cell sorting of CD45.2^+^ GC B cells at both day 8 and day 22. Despite full-length PfCSP containing junctional and minor repeat regions, no iGL_CIS43 cells were detected from that group, and few iGL_L9 cells were observed (2/894 on day 8 and 1/270 on day 22). GC responses were instead dominated by the two anti-major repeat clones, iGL_311 and iGL_317 (Fig. 7 F, left). Truncated PfCSP exclusively induced iGL_311 responses at day 8 and yielded no sequences at day 22 due to the scarcity of CD45.2^+^ GC B cells (Fig. 7 F, center). In contrast, truncated PfCSP + peptide-KLH immunogens to the minor and junctional regions activated all three epitope-specific B cell populations. During the early GC phase, anti-major and anti-minor repeat clones dominated, whereas by the late phase, anti-junctional and anti-major clones were more prevalent (Fig. 7 F, right).

To investigate the affinity maturation of each precursor, we analyzed their mutations and compared them with the corresponding mature Ab sequences to identify on-track mutations from CD45.2^+^ GC B cells in the truncated PfCSP + peptides immunization group at day 22. In iGL_311, a significant S31N mutation in HCDR1—critical for its homotypic interaction—was observed. Additional on-track mutations, including Y59F near HCDR2 and S100T in HCDR3, were also frequently induced. In iGL_CIS43, on-track mutations such as N52K and K59R in HCDR2—key residues for binding to the junctional epitope—were induced at 6.8 and 64%, respectively, along with M34I in HCDR1 and V98L in HCDR3. This mutation pattern closely mirrored our previous findings for iGL_CIS43 (Kratochvil et al., 2021). In iGL_L9, on-track mutations including T28I and S31T in HCDR1, Y53F in HCDR2, and Y96F in HCDR3 were induced, consistent with results from single-clone transfer experiments (Fig. 7 G). Collectively, these data confirm that upon truncated PfCSP + peptide-KLH immunization, each clone acquired on-track mutations supporting affinity maturation toward their corresponding mature Abs despite a competitive co-adoptive transfer environment.

Next, to assess the polyclonal response, including endogenous host responses, we performed serum ELISA analysis using day 22 samples. NANP18, NPDP19, and Pep22 peptides were used to detect anti-major repeat, anti-junctional, and anti-minor repeat responses, respectively. Serum from mice immunized with full-length PfCSP elicited stronger anti-major repeat Ab responses than the truncated PfCSP + peptides group; truncated PfCSP alone fell between these groups. However, anti-junctional and anti-minor repeat responses were significantly higher in the truncated PfCSP + peptides group (Fig. 7 H). In sum, the addition of peptide-KLH immunogens improved junctional and minor repeat Ab responses.

### Immunofocusing peptide induces substantial anti-parasite protection in vivo

Finally, we tested whether the anti-junctional and anti-minor repeat epitope Abs elicited by the peptide-KLHs could confer improved protection. The current R21/Matrix-M immunization regimen is three doses given 1 mo apart followed by a fourth dose 12 mo after the third. To mimic this longer interval between prime and boost, adoptively co-transferred mice were primed and then boosted at 4–5 mo (Fig. 7 I).

We first compared serum IgG titers before and after the boost. In adoptive transfer recipients, immunization by truncated PfCSP + peptides did not diminish anti-major responses relative to immunization by PfCSP or truncated CSP alone; PfCSP + peptide immunization did, however, significantly increase anti-junction and anti-minor repeat titers; these responses, notably, were observed 3 days pre-boost, 4–5 mo after the initial prime, indicating a durable response to the prime. All titers were elevated after each boost (Fig. 7 J). Immunized mice were then challenged with live *P*. *berghei* expressing PfCSP 10 days post-boost, and liver burden was assessed 2 days after challenge (Fig. 7 I). Despite the anti-major IgG titers elicited by all immunizations, significant protective efficacy was observed only in the truncated CSP + peptides group; no significant reduction in liver burden was obtained by any other immunization protocol (Fig. 7 K). Thus, the addition of immunofocusing peptides broadened the Ab response and provided antimalarial protection in vivo.

## Discussion

The recently approved malaria vaccines R21 and RTS,S have shown protective efficacy in infants and young children. Both vaccines mediate protection by inducing Abs against the major NANP repeat epitopes of PfCSP. High anti-NANP titers are required, and protective efficacy is limited relative to other childhood vaccines. Moreover, protection is not durable (Miura et al., 2024). Thus, expanding the breadth of the humoral repertoire to other neutralizing epitopes may be key to effective and long-lived malaria vaccines. Here, in murine models with B cells expressing human BCR precursors to highly protective Abs, we confirmed that an immunogen containing the truncated PfCSP epitopes presented by R21 cannot effectively activate antimalaria B cell precursors to anti-minor repeat or junctional region Abs. Furthermore, the immune response to full-length PfCSP, which includes junctional and minor epitopes, was predominantly directed toward major repeats and was inadequate to elicit stable anti-minor GC responses. An immunofocusing strategy using epitope-specific short peptides, however, induced robust GC responses from both low- and high-affinity anti-minor repeat (L9) precursors. Furthermore, this approach facilitated the affinity maturation of high-affinity anti-minor repeat B cells—suggesting that we may achieve improved protective efficacy even in malaria-experienced populations and allowing us to assess the sequence and structural correlates of anti-minor repeat Ab protection. Finally, by combining the R21-epitope truncated PfCSP with minor and junctional epitope peptide-KLH immunogens, we elicited a balanced, protective humoral response in vivo, suggesting that targeting additional epitopes may enhance current malaria vaccines.

Traditionally, antigen immunogenicity has been evaluated using WT mice. However, murine immune repertoires may not accurately represent those of humans, necessitating BCR-transgenic models. Building upon our previous investigation into the PfCSP junctional epitope (Kratochvil et al., 2021), this study has advanced our preclinical mouse platform by incorporating human Ab precursors of additional PfCSP specificities. Genetically modified mice in which B cells bear human BCRs have been instrumental in HIV vaccinology, facilitating several successful phase 1 clinical trials. The efficacy of the BG505 SOSIP.v4.1-GT1.1 immunogen in phase 1 (Caniels et al., 2025) corresponded well with the prediction of immunogenicity of GT1 variants obtained in genetically modified mice (Caniels et al., 2023; Caniels et al., 2024; Medina-Ramírez et al., 2017). Similarly, the ability of the eOD-GT8 60mer immunogen to activate rare B cell precursors in humans (Leggat et al., 2022) was foreshadowed by its performance in Ig KI mice similar to those used here (Abbott et al., 2018; Dosenovic et al., 2015; Huang et al., 2020; Jardine et al., 2015). The presence of human-derived HC and LC sequences allows for tracking not only B cell activation and differentiation but also the acquisition of specific mutations associated with affinity and breadth. The lines developed here and previously by our group (Kratochvil et al., 2021) allow for the expansion of these genetic technologies to malaria therapeutic and vaccine development. While some results from our new PfCSP mouse models were consistent with observed affinities, Ab-antigen affinity measurements alone were not fully predictive of in vivo outcomes: of note, iGL_311 has higher affinity than iGL_317 to the major repeats, minor repeats, and the junctional region, yet iGL_317 activation after full-length PfCSP immunization was far stronger. Structural features of the BCR–antigen interaction on the B cell surface—including receptor clustering and signaling threshold—may differ between the two clones in ways not captured by solution-phase BLI measurements. These observations, and the collective body of work, underscore the importance of in vivo mouse models with human-derived BCR sequences for immunogenicity studies.

Congenic adoptive transfer from models with pre-rearranged B cells bearing BCRs of known specificities enables the establishment of calibrated precursor frequencies within an intact murine repertoire. Alternative approaches, such as recombining mouse models (Tian et al., 2016) and Kymab mice (Sok et al., 2016), are also available and have contributed to the development of candidate HIV immunogens. However, early recombining mice had an unphysiologically high number of relevant precursors, while Kymab mice averaged only 1.3 precursors per animal for eOD-GT8, thus underestimating its later performance. More recent rearranging models have thus been designed to produce more physiological frequencies (Luo et al., 2023). As precursor frequency is a crucial determinant of immunogenicity (Abbott et al., 2018; Dosenovic et al., 2018), the ability to precisely titrate frequencies is critical to replicate human physiology. Although the precise frequencies of anti-major, anti-minor, and anti-junction precursors have not yet been determined in the human population, the extremely low frequencies we deployed here make the new anti-major and anti-minor repeat mouse models described here quite stringent.

The flexibility offered by adoptive transfer from multiple KI lines into a single congenic host enabled the examination of the relationship between protective efficacy and combined B cell immune responses. Although both full-length PfCSP and truncated PfCSP induced activation of anti-major iGL_311 and iGL_317 precursors, these responses did not translate into significant protective efficacy. The avidity of the genuine R21 vaccine presentation, as opposed to the monomer here, may affect this response. Alternatively, the lack of protection may be due to the timeline: the 4+ month gap after the prime may have allowed anti-major titers to wane too substantially to be protective on their own. Finally, as the skin is the locus of activity for some anti-central–repeat Abs (Aliprandini et al., 2018), mosquito-bite models of protection may have a lower protective threshold for similar Abs. However, it is clear that after defined IV delivery, achieving robust protection required inclusion of anti-junction (iGL_CIS43) and anti-minor (iGL_L9) responses. In addition, anti-junction and anti-minor IgG titers were durable and were significantly boosted 4–5 mo after priming, suggesting that peptide-KLH immunogens can elicit substantial long-lived plasma cell and memory B cell responses even after a single immunization. Together, these findings demonstrate that our model is a robust platform for evaluating the protective potential of malaria vaccine candidates and identifying B cell clones that are functionally associated with protection.

As several potent Abs against the junctional and minor repeat regions have been reported, and as B cells targeting the same epitope may compete and limit antigen accessibility (Dvorscek et al., 2024; Schaefer-Babajew et al., 2023; Tas et al., 2022), eliciting neutralizing Abs against diverse PfCSP epitopes may improve protective efficacy. Although full-length PfCSP contains epitopes capable of activating L9 or CIS43 precursors, it failed to sustain a stable GC response. Truncated PfCSP (R21-epitope) also failed to elicit these protective Abs at all, although the responding B cell repertoire in actual vaccination may differ due to avidity effects, since, in contrast to the R21-epitope monomeric truncated PfCSP used in this study, R21 is a HBsAg-linked multimeric immunogen (Collins et al., 2017). In contrast, our epitope-specific peptide-KLH multimer immunogen design increases the molecular abundance of junctional and minor repeat epitopes relative to PfCSP, reducing competitive suppression. A single immunization with peptide-KLH immunogens alongside the R21-epitope truncated PfCSP was sufficient to elicit a significant GC response and affinity maturation in anti-junction and anti-minor B cells, while maintaining a response to the major repeats. Recent reports have also described vaccine designs specifically targeting anti-junctional and anti-minor repeat B cells (Francica et al., 2021; Jelínková et al., 2021; Jelínková et al., 2022; Langowski et al., 2025; Ludwig et al., 2023; Tripathi et al., 2026), collectively highlighting the importance of eliciting responses against these regions. The use of a Pep22-KLH boost also enhanced anti-minor repeat responses in our system. Abs generated during the primary immune response to PfCSP have been observed to mask the major repeat region during subsequent responses (McNamara et al., 2020); because the junctional and minor repeat regions also contain NANP sequences, the primary response may also interfere with anti-junctional and anti-minor repeat responses upon secondary immunization. Consequently, extended or repeat exposures to PfCSP may be required to expand past the major repeat–biased response; the supraphysiological doses of irradiated PfSPZ that successfully isolated these anti-minor and anti-major Abs may fall in this category (Kisalu et al., 2018; Wang et al., 2020). The application of immunofocusing to boost immunogen cocktails may also avert this major repeat bias.

Vaccination is not the only mechanism of antimalarial prophylaxis, as evinced by the clinical trials of L9LS and CIS43LS (Lyke et al., 2023; Wu et al., 2022; Kayentao et al., 2022; Kayentao et al., 2024; Gaudinski et al., 2021). We anticipated that variants with higher affinity than mature L9 would exhibit greater in vivo potency and that a significant decrease in affinity (compared with L9) would be associated with a clear reduction in in vivo potency; indeed, most of the variants we identified with affinity two to four times higher than L9 exhibited similar levels of in vivo potency. However, the correlations we observed between affinity and protection appeared to hold only when assessed over a large range. Thus, while lower affinity than that of L9 often showed reduced protection, higher affinities did not guarantee increased protection in vivo. Instead, the specific impact of the variant mutation on PfCSP binding in the context of the SPZ is likely more critical for in vivo efficacy; this aligns with previous findings where higher affinity did not correlate with increased in vivo potency for major repeat Abs that exhibit high in vivo potency (Williams et al., 2024), although improved efficacy could result from carefully selected junctional region Abs (Banach et al., 2022; Reveiz et al., 2025). With the L9 variants, we analyzed on-rate/off-rates to see if we could gain insight into how affinity related to improved protection. However, the L9 variants with significantly higher affinity did not display significantly higher protective efficacy than L9. Therefore, further improvement would require not only research on the relationship between affinity and protection, but also the structural changes induced by Abs in PfCSP, and their relationship with liver cell invasion and attachment.

Cross-reactivity against the major repeat, minor repeat, and junctional region may be a key factor for protective efficacy. The current RTS,S and R21 vaccines were designed based on the finding that Abs targeting the major repeat region exhibit protective efficacy, and the level of anti-major repeat Abs in the serum has shown correlation with clinical efficacy (Datoo et al., 2024). However, Abs recognizing both the major repeat and either the minor repeat or junctional regions have higher protective efficacy than those recognizing only the major repeat (Murugan et al., 2020). Notably, R21 vaccination has been shown to elicit Abs that cross-react with the junctional region despite the absence of this epitope in the immunogen, suggesting that recipients of the R21 vaccine retain the capacity to mount high-affinity responses against non-targeted epitopes (McDaniel et al., 2025). Consistent with this broader recognition potential, L9, as observed through ITC analysis, exhibited two-step binding to PfCSP, indicating an affinity for both the minor and major repeat regions. F10, an Ab that shares a common ancestor with L9, shows a lower affinity for the major repeat region and significantly lower in vivo protection efficacy (Wang et al., 2022). The mechanism underlying the superior efficacy of Abs targeting both regions remains unclear; however, the affinity for the major repeat region likely provides a strong binding effect owing to the avidity of the repeated sequences, facilitating PfCSP recognition. Just ahead of the junctional region is a region rich in positively charged AA, which plays a crucial role in heparan sulfate proteoglycan (HSPG) binding and is expected to be important for the role of PfCSP in liver invasion (Ancsin and Kisilevsky, 2004; Coppi et al., 2007; Frevert et al., 1993); Abs against the junctional or minor repeat regions may therefore interfere with HSPG binding, thereby preventing liver invasion, although this requires experimental validation.

The addition of short peptide-based immunofocusing immunogens may enhance the suboptimal vaccine efficacy of PfCSP epitopes included in R21 by activating a broader antimalaria response. In a previous study, immunization with R21 followed by passive transfer of anti-junctional or anti-minor repeat Abs exhibited added efficacy in an in vivo challenge model (Wang et al., 2021a) and a recent study demonstrated that tandem delivery of a CIS43-based junctional vaccine in mice improved protection at low doses of R21 (Tripathi et al., 2026); we might therefore expect synergy between R21 and immunofocusing immunogens. Given the variability of the human B cell clonal landscape, controlled human malaria infection studies would be the next logical step to validate these findings. Overall, this study demonstrates that including the junctional and minor repeat PfCSP epitopes, potentially in the form of immunofocusing peptides, may enhance the current generation of malaria vaccines.

## Materials and methods

### BCR KI mouse model

iGL_L9, L9, iGL_311, and iGL_317 BCR-KI (both HC and LC) mice were generated at the animal facility of the Gene Modification Facility (Harvard University) using methods we previously published (Lin et al., 2018; Wang et al., 2021b). Subsequent colony breeding and experiments were conducted at the Ragon Institute of Mass General Brigham, MIT, and Harvard. Seven-to nine-week-old male B6.SJL-Ptprca Pepcb/BoyJ CD45.1 host mice (RRID:IMSR_JAX:002014) were purchased from The Jackson Laboratory, acclimated for 1 wk, and then used for adoptive transfer experiments. All animal experiments were conducted in accordance with protocols (2016N000286 and 2016N000022) approved by the Institutional Animal Care and Use Committee (IACUC) of Harvard University and the Massachusetts General Hospital, an Association for Assessment and Accreditation of Laboratory Animal Care (AAALAC) international accredited facility.

### Adoptive transfer model and immunization

B cells were isolated from CD45.2 BCR KI mice using the Pan B Cell Isolation Kit II (Miltenyi Biotec). The isolated B cells were stained with probes and Abs, and precursor frequency was calculated using a BD FACSymphony S6. The B cells were then diluted in PBS to a volume of 200 μl and adoptively transferred into B6.SJL-Ptprca Pepcb/BoyJ host mice (CD45.1) via tail vein injection. One day later, the mice were immunized. Sample sizes were derived from prior work with this model type (Kratochvil et al., 2021); no mice were excluded from analysis.

Protein or Peptide-KLH antigen (GenScript), 10 mg, was diluted in 100 μl of PBS and mixed with 2% Alhydrogel (InvivoGen) in a 1:1 volume ratio. This mixture was incubated on a rotator for 30 min and then administered intraperitoneally to mice (10 µg in 200 μl per mouse). All animal experiments were conducted in accordance with the approved protocols by the IACUC of Harvard University and Massachusetts General Hospital.

### Flow cytometry

Individual spleens were ground on a 70-µm filter to isolate single cells, followed by the removal of RBCs using ACK lysis buffer. The RBC-free cells were then incubated with Live/Dead Blue and FC block, diluted in PBS containing 2% FBS (FACS buffer), at 4°C for 10 min and subsequently washed. The biotinylated probe was first incubated with fluorochrome-streptavidin at a 4:1 M ratio at 4°C for 20 min, then diluted in FACS buffer to a probe concentration of 50 nM and incubated with the cells at 4°C for 30 min before washing. For surface staining, Abs were diluted in FACS buffer at a ratio of 1:100 to 1:400 and incubated with the cells at 4°C for 30 min, followed by two washes. The prepared cells were analyzed or sorted using a BD FACSymphony S6. Single cells were dry-sorted into a 96-well PCR plate, quickly frozen on dry ice, and stored in a −80°C deep freezer for future sequencing. FACS data were analyzed using FlowJo10.

### BCR sequencing

First, cDNA synthesis was performed using SuperScript III Reverse Transcriptase (Invitrogen) according to the manufacturer’s instructions. Subsequently, HC and LC PCRs were conducted separately, each performed twice in a nested format. The PCR was carried out using 10× PCR buffer (QIAGEN), HotStarTaq enzyme (QIAGEN), 10 mM dNTPs (Thermo Fisher Scientific), and a cocktail of IgG or IgK-specific primer sets (von Boehmer et al., 2016) diluted in water. The PCR products were electrophoresed on E-Gels 96 2% with SYBR Safe (Thermo Fisher Scientific), and the PCR bands were visualized using a ChemiDoc imaging system (Bio-Rad). Following this, the PCR plate was sent to Genewiz, where Sanger sequencing was performed using the HC reverse primer from PCR-2 (5′-GCTCAGGGAARTAGCCCTTGAC-3′) and the LC reverse primer (5′-TGG​GAA​GAT​GGA​TAC​AGT​T-3′) to confirm the BCR sequence.

For the mixed adoptive transfer model, single-cell RNA sequencing was performed by generating NGS libraries from sorted cells with the Chromium Next GEM Single Cell 5′ Reagent Kit v2 (10x Genomics), following the manufacturer’s protocol. The libraries were pooled and sequenced on a NextSeq 2000 instrument (Illumina). Sequencing reads were processed and analyzed using Cell Ranger software (version 8, 10x Genomics) with a customized reference database. Sequencing data were excluded if they failed standard quality thresholds, such as multiple peaks in Sanger sequencing.

### ELISA

96-well plates were coated with 25 μl/well of PfCSP (1 µg/ml) diluted in PBS, and incubated overnight at 4°C. The next day, the coating solution was discarded, and plates were washed three times with 0.05% Tween 20 in PBS (tPBS). 100 μl/well of blocking buffer (tPBS with 3% BSA) was added prior to incubation for 1 h at room temperature on an orbital shaker. The blocking solution was discarded, and the plates were washed three times with tPBS. Mouse serum diluted (1:150) in tPBS with 0.5% BSA was then added (60 μl/well), and a threefold serial dilution was performed prior to incubation overnight at 4°C. The next day, the unreacted solution was discarded, and plates were washed three times with tPBS. Secondary AP-conjugated anti-mouse IgG Ab (Cat# 115-055-003; Jackson ImmunoResearch Labs; RRID: AB_2338528) diluted 1:1,000 in tPBS with 0.5% BSA was added (50 μl/well) and plates incubated for 1 h at room temperature on an orbital shaker. Unreacted Ab solution was discarded, and plates washed five times with 100 μl tPBS and once with dH_2_O. pNPP dissolved in dH2O was added (50 μl/well) and plates incubated for 30 min at room temperature. The reaction was stopped by adding 50 μl of 3N NaOH. Optical density was measured at 450 nm using a BioTek Synergy Neo2 microplate reader (Agilent).

### Bead assay

Protein A beads (10 μl) were diluted in 1 ml of PBS and washed. Then, 1 µg of Ab was diluted in PBS to a total volume of 50 μl and incubated with the beads for 30 min at room temperature to capture the Ab. Afterward, 0.5 µg of rat IgG2c isotype control Ab (Cat# 400701; BioLegend; RRID:AB_326567) was added and incubated for 10 min at room temperature to block nonspecific binding. The beads were washed with PBS, then incubated with 10 nM of the prepared probe for 30 min at room temperature to allow probe binding. Following two washes, the probe binding intensity of the Ab-captured beads was analyzed using a BD FACSymphony S6. FACS data were analyzed using FlowJo10.

### Ab expression and purification

Ab variable HC and LC sequences were codon optimized, synthesized, and cloned into a VRC8400 (CMV/R expression vector)-based IgG1 vector as previously described (Kong et al., 2019). The variants were expressed by transient transfection in Expi293 cells (Thermo Fisher Scientific) using Turbo293 transfection reagent (SPEED BioSystems) according to the manufacturer’s recommendation. 50 μg of plasmid encoding HC and 50 μg of plasmid encoding LC variant genes were mixed with the transfection reagents, added to 100 ml of cells at 2.5 × 10^6^/ml, and incubated in a shaker incubator at 120 rpm, 37°C, 9% CO_2_. At 5 days after transfection, cell culture supernatant was harvested and purified with a Protein A (GE Healthcare) column. The Ab was eluted using IgG Elution Buffer (Thermo Fisher Scientific) and were brought to neutral pH with 1 M Tris-HCl, pH 8.0. Eluted Abs were dialyzed against PBS overnight before use.

### Cryo-EM sample preparation, grid preparation, and data collection

The sample for cryo-EM was prepared by mixing Fab to PfCSPm at 2:1 M ratio. The complex was incubated overnight at 4°C and flash frozen until ready for use. To prepare grids of the complex, 3 μl of protein sample was applied to freshly glow-discharged (easiGlow) C-flat grids (Protochips, CF1.2/1.3-3Au). Blotting was done using a Vitrobot Mark IV (Thermo-Fisher), with a 5-s blotting time and 8 pN blotting force at 6°C in 100% humidity. Grids were vitrified by plunging into liquid ethane and stored in liquid nitrogen before examination by cryo-EM. Images were recorded on a Glacios TEM (Thermo Fisher Scientific) at 200 kV and recorded at 36,000× magnification with a defocus range of −0.3 to −2.2 µm on K3 direct electron detector (Gatan) in super-resolution mode.

### Cryo-EM data processing and refinement

Motion correction, contrast transfer function estimation, particle picking, extraction, 2D classification, ab initio model generation, 3D refinements, and local resolution estimation were carried out in cryoSPARC 3.3.1 (RRID:SCR_016501) (Punjani et al., 2017). The 3D reconstructions were performed using C1 symmetry for both classes. The coordinates of the L9-PfCSP structure, PDB entry (8EK1), were employed as an initial model for fitting the sharpened cryo-EM map of the L9-PfCSP structures (Table S2). Manual and automated model building were iteratively performed using Coot (RRID:SCR_014222) (Emsley and Cowtan, 2004) and real space refinement in Phenix (Adams et al., 2010) to accurately fit the coordinates to the electron density map. MolProbity (RRID:SCR_014226) (Davis et al., 2004) and EMRinger (Barad et al., 2015) were used to validate geometry and check structure quality. UCSF ChimeraX (Goddard et al., 2018) was used for map-fitting cross-correlation calculation (Fit-in-Map tool) and for figure preparation.

### Kinetics-guided Ab design

Variants were designed iteratively based on BLI measurements of association (kon) and dissociation (koff) rates against PfCSP and peptide antigens. Initial L9 variants (including day 28 isolates) were profiled to identify mutations associated with slower dissociation and/or faster association. Mutations showing anticorrelation with protection, particularly at peptide-contact or homotypic Fab–Fab interface positions, were deprioritized. In the first round, mutations enriched among higher-performing variants were recombined onto selected templates (L9-11, L9-32, and L9-21) to generate combination designs. In the second round, L9-com16, selected based on balanced kinetic improvements, was used as the template for further optimization by introducing additional mutations associated with favorable kinetic profiles. All variants were expressed and screened by BLI, and those with improved kinetics were advanced for further characterization.

### Affinity measurements by BLI

Ab binding affinity to various ligands was measured using BLI on an Octet Red384 instrument (FortéBio) with streptavidin capture biosensors (FortéBio) in solid black tilt-well 96-well plates (Geiger Bio-One). Assays were performed with agitation at 30°C. Biotinylated peptides were diluted to 0.1 μg/ml in (PBS + 1% BSA) and were immobilized for 3 s to reduce the density (<0.01 nm) on the biosensor to avoid avidity. This step was followed by a 60-s baseline in buffer (PBS + 1% BSA). Association with IgG (serially diluted from 1,000 to 1.3 nM was done for 240 s, followed by a dissociation step in buffer for 1,200 s. In all Octet measurements, parallel correction to subtract systematic baseline drift was carried out by subtracting the measurements recorded for a loaded sensor incubated in PBS. Data analysis was carried out using Octet software, version 9.0. Experimental data were fitted globally with a 1:1 Langmuir model of binding for all the antigens.

### Animals for parasite challenge

Female C57Bl/6 mice (RRID: IMSR_JAX:000664) of 6–8 wk of age were purchased from The Jackson Laboratories. All animals were maintained and cared for in accordance with AAALAC Standards. All studies were approved by the Animal Care and Use Committee at Johns Hopkins University (JHU), protocol numbers MO21H417 and MO24H329.

### Parasites

Transgenic SPZ in *P. berghei* expressing PfCSP, GFP, and luciferase reporter gene, used in all studies, have been previously described (Flores-Garcia et al., 2019). Briefly, 5-day-old adult *Anopheles stephensi* mosquitoes (maintained at the insectary at JHU, Malaria Research Institute) were allowed to feed on Swiss Webster mice carrying 2–3% parasitemia. Then, 20–22 days after murine blood meal, transgenic SPZ were collected from salivary glands and used within 60 min for IV infection for liver burden studies.

### Reduction in liver infection assay

To assess the protective efficacy of anti-PfCSP mAbs in vivo*,* mAbs were diluted in sterile PBS at the desired dilution. Mice were then passively immunized IV in the tail vein. 2 h later, animals were challenged IV with 2,000 *P. berghei* transgenic SPZ that express full-length PfCSP and GFP-Luc enzyme. 42 h after parasite challenge, parasite load in the liver was measured by bioluminescence in an in vivo imaging system (IVIS Spectrum, Perkin Elmer). Mice were injected intraperitoneally with 100 μl of d-luciferin (30 mg/ml) and immediately anesthetized with isoflurane for 5 min prior to IVIS. Groups of five anesthetized mice were placed in the imager, and the radiance measurements were recorded by the live imager software, version 4.5.1. The total flux reading for each mouse was recorded individually. Background reading was verified for each study with mice that received only the d-luciferin substrate.

### Statistical analysis

Significant differences were calculated with Mann–Whitney’s *t* test (between two groups) assuming non-normal distribution, one-way, or two-way ANOVA (among three or more groups) with Tukey’s multiple-comparisons test applied throughout. P values are shown as no significant difference (ns) P > 0.05, *P < 0.05, **P < 0.01, ***P < 0.001, and ****P < 0.0001. All P value analyses were calculated using GraphPad Prism 10 (RRID: SCR_002798).

### Online supplemental material

Fig. S1 establishes adoptive transfer precursor frequencies and relates to Fig. 1. Fig. S2 shows extended iGL_L9 and L9 B cell responses to immunogens, related to Fig. 2. Fig. S3 shows the SHM and affinity of Ab variants produced by those responses, related to Fig. 3. Fig. S4 provides additional in vivo and biophysical characterization of postimmunization L9-derived Abs, related to Fig. 4, 5, and 6. Fig. S5 provides supporting details for cryo-EM, related to Fig. 5 and Table S2. Table S1 details L9-variant mAb expression, and Table S2 details cryo-EM statistics and deposition information.

## Supplementary Material

Table S1 shows expression of L9 variant mAbs.

Table S2 shows cryo-EM single-particle data collection and refinement statistics.

## Data Availability

KI mouse models are available from F.D. Batista on request under a standard material transfer agreement with Mass General Hospital. BCR sequences are available at GenBank: PZ498966–PZ499037 and PZ498895–PZ498965. PDB deposition numbers are listed in Table S2.

## References

[bib1] Abbott, R.K., J.H. Lee, S. Menis, P. Skog, M. Rossi, T. Ota, D.W. Kulp, D. Bhullar, O. Kalyuzhniy, C. Havenar-Daughton, . 2018. Precursor frequency and affinity determine B cell competitive fitness in germinal centers, tested with germline-targeting HIV vaccine immunogens. Immunity. 48:133–146.e6. 10.1016/j.immuni.2017.11.023 29287996 PMC5773359

[bib2] Adams, P.D., P.V. Afonine, G. Bunkóczi, V.B. Chen, I.W. Davis, N. Echols, J.J. Headd, L.-W. Hung, G.J. Kapral, R.W. Grosse-Kunstleve, . 2010. PHENIX: A comprehensive python-based system for macromolecular structure solution. Acta Crystallogr. D Biol. Crystallogr. 66:213–221. 10.1107/S0907444909052925 20124702 PMC2815670

[bib3] Aliprandini, E., J. Tavares, R.H. Panatieri, S. Thiberge, M.M. Yamamoto, O. Silvie, T. Ishino, M. Yuda, S. Dartevelle, F. Traincard, . 2018. Cytotoxic anti-circumsporozoite antibodies target malaria sporozoites in the host skin. Nat. Microbiol. 3:1224–1233. 10.1038/s41564-018-0254-z 30349082

[bib4] Ancsin, J.B., and R. Kisilevsky. 2004. A binding site for highly sulfated heparan sulfate is identified in the N terminus of the circumsporozoite protein. J. Biol. Chem. 279:21824–21832. 10.1074/jbc.M401979200 15007056

[bib5] Banach, B.B., P. Tripathi, L. Da Silva Pereira, J. Gorman, T.D. Nguyen, M. Dillon, A.S. Fahad, P.K. Kiyuka, B. Madan, J.R. Wolfe, . 2022. Highly protective antimalarial antibodies via precision library generation and yeast display screening. J. Exp. Med. 219:e20220323. 10.1084/jem.20220323 35736810 PMC9242090

[bib6] Barad, B.A., N. Echols, R.Y.-R. Wang, Y. Cheng, F. DiMaio, P.D. Adams, and J.S. Fraser. 2015. EMRinger: Side chain-directed model and map validation for 3D cryo-electron microscopy. Nat. Methods. 12:943–946. 10.1038/nmeth.3541 26280328 PMC4589481

[bib7] Caniels, T.G., M. Medina-Ramírez, J. Zhang, A. Sarkar, S. Kumar, A. LaBranche, R. Derking, J.D. Allen, J.L. Snitselaar, J. Capella-Pujol, . 2023. Germline-targeting HIV-1 Env vaccination induces VRC01-class antibodies with rare insertions. Cell Rep. Med. 4:101003. 10.1016/j.xcrm.2023.101003 37044090 PMC10140475

[bib8] Caniels, T.G., M. Medina-Ramìrez, S. Zhang, S. Kratochvil, Y. Xian, J.-H. Koo, R. Derking, J. Samsel, J. van Schooten, S. Pecetta, . 2024. Germline-targeting HIV vaccination induces neutralizing antibodies to the CD4 binding site. Sci. Immunol. 9:eadk9550. 10.1126/sciimmunol.adk9550 39213338 PMC11783328

[bib9] Caniels, T.G., M. Prabhakaran, G. Ozorowski, K.J. MacPhee, W. Wu, K. van der Straten, S. Agrawal, R. Derking, E.I.M.M. Reiss, K. Millard, . 2025. Precise targeting of HIV broadly neutralizing antibody precursors in humans. Science. 389:eadv5572. 10.1126/science.adv5572 40373114 PMC12313413

[bib10] Cockburn, I.A., and R.A. Seder. 2018. Malaria prevention: From immunological concepts to effective vaccines and protective antibodies. Nat. Immunol. 19:1199–1211. 10.1038/s41590-018-0228-6 30333613

[bib11] Collins, K.A., S.C. Gilbert, and A.V.S. Hill. 2017. Assignee: Oxford University Innovation Ltd. Composition and uses thereof. United States Patent no. US9821046B2, filed January 21, 2014, and issued November 21, 2017.

[bib12] Coppi, A., R. Tewari, J.R. Bishop, B.L. Bennett, R. Lawrence, J.D. Esko, O. Billker, and P. Sinnis. 2007. Heparan sulfate proteoglycans provide a signal to *Plasmodium* sporozoites to stop migrating and productively invade host cells. Cell Host Microbe. 2:316–327. 10.1016/j.chom.2007.10.002 18005753 PMC2117360

[bib13] Datoo, M.S., A. Dicko, H. Tinto, J.-B. Ouédraogo, M. Hamaluba, A. Olotu, E. Beaumont, F.R. Lopez, H.M. Natama, S. Weston, . 2024. Safety and efficacy of malaria vaccine candidate R21/Matrix-M in African children: A multicentre, double-blind, randomised, phase 3 trial. Lancet. 403:533–544. 10.1016/S0140-6736(23)02511-4 38310910 PMC7618965

[bib14] Davis, I.W., L.W. Murray, J.S. Richardson, and D.C. Richardson. 2004. MOLPROBITY: Structure validation and all-atom contact analysis for nucleic acids and their complexes. Nucleic Acids Res. 32:W615–W619. 10.1093/nar/gkh398 15215462 PMC441536

[bib15] Dosenovic, P., E.E. Kara, A.-K. Pettersson, A.T. McGuire, M. Gray, H. Hartweger, E.S. Thientosapol, L. Stamatatos, and M.C. Nussenzweig. 2018. Anti-HIV-1 B cell responses are dependent on B cell precursor frequency and antigen-binding affinity. Proc. Natl. Acad. Sci. USA. 115:4743–4748. 10.1073/pnas.1803457115 29666227 PMC5939114

[bib16] Dosenovic, P., L. von Boehmer, A. Escolano, J. Jardine, N.T. Freund, A.D. Gitlin, A.T. McGuire, D.W. Kulp, T. Oliveira, L. Scharf, . 2015. Immunization for HIV-1 broadly neutralizing antibodies in human Ig knockin mice. Cell. 161:1505–1515. 10.1016/j.cell.2015.06.003 26091035 PMC4604566

[bib17] Dvorscek, A.R., C.I. McKenzie, V.C. Stäheli, Z. Ding, J. White, S.A. Fabb, L. Lim, K. O’Donnell, C. Pitt, D. Christ, . 2024. Conversion of vaccines from low to high immunogenicity by antibodies with epitope complementarity. Immunity. 57:2433–2452.e7. 10.1016/j.immuni.2024.08.017 39305904

[bib18] Emsley, P., and K. Cowtan. 2004. Coot: Model-building tools for molecular graphics. Acta Crystallogr. D Biol. Crystallogr. 60:2126–2132. 10.1107/S0907444904019158 15572765

[bib19] Enea, V., J. Ellis, F. Zavala, D.E. Arnot, A. Asavanich, A. Masuda, I. Quakyi, and R.S. Nussenzweig. 1984. DNA cloning of *Plasmodium falciparum* circumsporozoite gene: Amino acid sequence of repetitive epitope. Science. 225:628–630. 10.1126/science.6204384 6204384

[bib20] Feng, G., L. Kurtovic, P.A. Agius, E.H. Aitken, J. Sacarlal, B.D. Wines, P.M. Hogarth, S.J. Rogerson, F.J.I. Fowkes, C. Dobaño, and J.G. Beeson. 2022. Induction, decay, and determinants of functional antibodies following vaccination with the RTS,S malaria vaccine in young children. BMC Med. 20:289. 10.1186/s12916-022-02466-2 36002841 PMC9402280

[bib21] Flores-Garcia, Y., S.M. Herrera, H. Jhun, D.W. Pérez-Ramos, C.R. King, E. Locke, R. Raghunandan, and F. Zavala. 2019. Optimization of an in vivo model to study immunity to *Plasmodium falciparum* pre-erythrocytic stages. Malar. J. 18:426. 10.1186/s12936-019-3055-9 31849326 PMC6918627

[bib22] Francica, J.R., W. Shi, G.-Y. Chuang, S.J. Chen, L. Da Silva Pereira, S.K. Farney, B.J. Flynn, L. Ou, T. Stephens, Y. Tsybovsky, . 2021. Design of alphavirus virus-like particles presenting circumsporozoite junctional epitopes that elicit protection against malaria. Vaccines. 9:272. 10.3390/vaccines9030272 33803622 PMC8003078

[bib23] Frevert, U., P. Sinnis, C. Cerami, W. Shreffler, B. Takacs, and V. Nussenzweig. 1993. Malaria circumsporozoite protein binds to heparan sulfate proteoglycans associated with the surface membrane of hepatocytes. J. Exp. Med. 177:1287–1298. 10.1084/jem.177.5.1287 8478608 PMC2190997

[bib24] Frischknecht, F., and K. Matuschewski. 2017. *Plasmodium* sporozoite biology. Cold Spring Harb. Perspect. Med. 7:a025478. 10.1101/cshperspect.a025478 28108531 PMC5411682

[bib25] Gardner, M.J., N. Hall, E. Fung, O. White, M. Berriman, R.W. Hyman, J.M. Carlton, A. Pain, K.E. Nelson, S. Bowman, . 2002. Genome sequence of the human malaria parasite *Plasmodium falciparum*. Nature. 419:498–511. 10.1038/nature01097 12368864 PMC3836256

[bib26] Gaudinski, M.R., N.M. Berkowitz, A.H. Idris, E.E. Coates, L.A. Holman, F. Mendoza, I.J. Gordon, S.H. Plummer, O. Trofymenko, Z. Hu, . 2021. A monoclonal antibody for malaria prevention. N. Engl. J. Med. 385:803–814. 10.1056/NEJMoa2034031 34379916 PMC8579034

[bib27] Goddard, T.D., C.C. Huang, E.C. Meng, E.F. Pettersen, G.S. Couch, J.H. Morris, and T.E. Ferrin. 2018. UCSF ChimeraX: Meeting modern challenges in visualization and analysis. Protein Sci. Publ. Protein Soc. 27:14–25. 10.1002/pro.3235 PMC5734306 28710774

[bib28] Gordon, D.M., T.W. McGovern, U. Krzych, J.C. Cohen, I. Schneider, R. LaChance, D.G. Heppner, G. Yuan, M. Hollingdale, M. Slaoui, . 1995. Safety, immunogenicity, and efficacy of a recombinantly produced *Plasmodium falciparum* circumsporozoite protein-hepatitis B surface antigen subunit vaccine. J. Infect. Dis. 171:1576–1585. 10.1093/infdis/171.6.1576 7769295

[bib29] Havenar-Daughton, C., R.K. Abbott, W.R. Schief, and S. Crotty. 2018. When designing vaccines, consider the starting material: the human B cell repertoire. Curr. Opin. Immunol. 53:209–216. 10.1016/j.coi.2018.08.002 30190230 PMC6148213

[bib30] Huang, D., R.K. Abbott, C. Havenar-Daughton, P.D. Skog, R. Al-Kolla, B. Groschel, T.R. Blane, S. Menis, J.T. Tran, T.C. Thinnes, . 2020. B cells expressing authentic naive human VRC01-class BCRs can be recruited to germinal centers and affinity mature in multiple independent mouse models. Proc. Natl. Acad. Sci. USA. 117:22920–22931. 10.1073/pnas.2004489117 32873644 PMC7502816

[bib31] Jardine, J.G., T. Ota, D. Sok, M. Pauthner, D.W. Kulp, O. Kalyuzhniy, P.D. Skog, T.C. Thinnes, D. Bhullar, B. Briney, . 2015. Priming a broadly neutralizing antibody response to HIV-1 using a germline-targeting immunogen. Science. 349:156–161. 10.1126/science.aac5894 26089355 PMC4669217

[bib32] Jelínková, L., Y. Flores-Garcia, S. Shapiro, B.T. Roberts, N. Petrovsky, F. Zavala, and B. Chackerian. 2022. A vaccine targeting the L9 epitope of the malaria circumsporozoite protein confers protection from blood-stage infection in a mouse challenge model. NPJ Vaccin. 7:34. 10.1038/s41541-022-00457-1 PMC8904524 35260593

[bib33] Jelínková, L., H. Jhun, A. Eaton, N. Petrovsky, F. Zavala, and B. Chackerian. 2021. An epitope-based malaria vaccine targeting the junctional region of circumsporozoite protein. Npj Vaccin. 6:1–10. 10.1038/s41541-020-00274-4 PMC7820318 33479242

[bib34] Julien, J.-P., and H. Wardemann. 2019. Antibodies against *Plasmodium falciparum* malaria at the molecular level. Nat. Rev. Immunol. 19:761–775. 10.1038/s41577-019-0209-5 31462718

[bib35] Kayentao, K., A. Ongoiba, A.C. Preston, S.A. Healy, S. Doumbo, D. Doumtabe, A. Traore, H. Traore, A. Djiguiba, S. Li, . 2022. Safety and efficacy of a monoclonal antibody against malaria in Mali. N. Engl. J. Med. 387:1833–1842. 10.1056/NEJMoa2206966 36317783 PMC9881676

[bib36] Kayentao, K., A. Ongoiba, A.C. Preston, S.A. Healy, Z. Hu, J. Skinner, S. Doumbo, J. Wang, H. Cisse, D. Doumtabe, . 2024. Subcutaneous administration of a monoclonal antibody to prevent malaria. N. Engl. J. Med. 390:1549–1559. 10.1056/NEJMoa2312775 38669354 PMC11238904

[bib37] Kisalu, N.K., A.H. Idris, C. Weidle, Y. Flores-Garcia, B.J. Flynn, B.K. Sack, S. Murphy, A. Schön, E. Freire, J.R. Francica, . 2018. A human monoclonal antibody prevents malaria infection by targeting a new site of vulnerability on the parasite. Nat. Med. 24:408–416. 10.1038/nm.4512 29554083 PMC5893371

[bib38] Kong, R., H. Duan, Z. Sheng, K. Xu, P. Acharya, X. Chen, C. Cheng, A.S. Dingens, J. Gorman, M. Sastry, . 2019. Antibody lineages with vaccine-induced antigen-binding hotspots develop broad HIV neutralization. Cell. 178:567–584.e19. 10.1016/j.cell.2019.06.030 31348886 PMC6755680

[bib39] Kratochvil, S., C.-H. Shen, Y.-C. Lin, K. Xu, U. Nair, L. Da Silva Pereira, P. Tripathi, J. Arnold, G.-Y. Chuang, E. Melzi, . 2021. Vaccination in a humanized mouse model elicits highly protective PfCSP-targeting anti-malarial antibodies. Immunity. 54:2859–2876.e7. 10.1016/j.immuni.2021.10.017 34788599 PMC9087378

[bib40] Langowski, M.D., J.R. Francica, A.L. Roederer, N.K. Hurlburt, J.V. Rodarte, L. Da Silva Pereira, B.J. Flynn, B. Bonilla, M. Dillon, P. Kiyuka, . 2025. Elicitation of liver-stage immunity by nanoparticle immunogens displaying *P. falciparum* CSP-derived antigens. NPJ Vaccin. 10:87. 10.1038/s41541-025-01140-x PMC12053698 40325041

[bib41] Leggat, D.J., K.W. Cohen, J.R. Willis, W.J. Fulp, A.C. deCamp, O. Kalyuzhniy, C.A. Cottrell, S. Menis, G. Finak, L. Ballweber-Fleming, . 2022. Vaccination induces HIV broadly neutralizing antibody precursors in humans. Science. 378:eadd6502. 10.1126/science.add6502 36454825 PMC11103259

[bib42] Lin, Y.-C., S. Pecetta, J.M. Steichen, S. Kratochvil, E. Melzi, J. Arnold, S.K. Dougan, L. Wu, K.H. Kirsch, U. Nair, . 2018. One-step CRISPR/Cas9 method for the rapid generation of human antibody heavy chain knock-in mice. EMBO J. 37:e99243. 10.15252/embj.201899243 30087111 PMC6138433

[bib43] Ludwig, J., S.W. Scally, G. Costa, S. Hoffmann, R. Murugan, J. Lossin, K. Prieto, A. Obraztsova, N. Lobeto, B. Franke-Fayard, . 2023. Glycosylated nanoparticle-based PfCSP vaccine confers long-lasting antibody responses and sterile protection in mouse malaria model. NPJ Vaccin. 8:52. 10.1038/s41541-023-00653-7 PMC10080175 37029167

[bib44] Luo, S., C. Jing, A.Y. Ye, S. Kratochvil, C.A. Cottrell, J.-H. Koo, A. Chapdelaine Williams, L.V. Francisco, H. Batra, E. Lamperti, . 2023. Humanized V(D)J-rearranging and TdT-expressing mouse vaccine models with physiological HIV-1 broadly neutralizing antibody precursors. Proc. Natl. Acad. Sci. USA. 120:e2217883120. 10.1073/pnas.2217883120 36574685 PMC9910454

[bib45] Lyke, K.E., A.A. Berry, K. Mason, A.H. Idris, M. O’Callahan, M. Happe, L. Strom, N.M. Berkowitz, M. Guech, Z. Hu, . 2023. Low-dose intravenous and subcutaneous CIS43LS monoclonal antibody for protection against malaria (VRC 612 Part C): A phase 1, adaptive trial. Lancet Infect. Dis. 23:578–588. 10.1016/S1473-3099(22)00793-9 36708738 PMC10121890

[bib46] MalariaGEN, M.M. Abdel Hamid, M.H. Abdelraheem, D.O. Acheampong, A. Ahouidi, M. Ali, J. Almagro-Garcia, A. Amambua-Ngwa, C. Amaratunga, L. Amenga-Etego, . 2023. Pf7: An open dataset of *Plasmodium falciparum* genome variation in 20,000 worldwide samples. Wellcome Open Res. 8:22. 10.12688/wellcomeopenres.18681.1 36864926 PMC9971654

[bib47] Martin, G.M., M.L. Fernández-Quintero, W.-H. Lee, T. Pholcharee, L. Eshun-Wilson, K.R. Liedl, M. Pancera, R.A. Seder, I.A. Wilson, and A.B. Ward. 2023. Structural basis of epitope selectivity and potent protection from malaria by PfCSP antibody L9. Nat. Commun. 14:2815. 10.1038/s41467-023-38509-2 37198165 PMC10192352

[bib48] McDaniel, J.R., W.N. Voss, G. Bowyer, S.A. Rush, A.J. Spencer, D. Bellamy, M. Ulaszewska, J. Goike, S. Gregory, C.R. King, . 2025. Repertoire, function, and structure of serological antibodies induced by the R21/Matrix-M malaria vaccine. J. Exp. Med. 222:e20241908. 10.1084/jem.20241908 40719751 PMC12302951

[bib49] McNamara, H.A., A.H. Idris, H.J. Sutton, R. Vistein, B.J. Flynn, Y. Cai, K. Wiehe, K.E. Lyke, D. Chatterjee, N. Kc, . 2020. Antibody feedback limits the expansion of B cell responses to malaria vaccination but drives diversification of the humoral response. Cell Host Microbe. 28:572–585.e7. 10.1016/j.chom.2020.07.001 32697938

[bib50] Medina-Ramírez, M., F. Garces, A. Escolano, P. Skog, S.W. de Taeye, I. Del Moral-Sanchez, A.T. McGuire, A. Yasmeen, A.-J. Behrens, G. Ozorowski, . 2017. Design and crystal structure of a native-like HIV-1 envelope trimer that engages multiple broadly neutralizing antibody precursors in vivo. J. Exp. Med. 214:2573–2590. 10.1084/jem.20161160 28847869 PMC5584115

[bib51] Miura, K., Y. Flores-Garcia, C.A. Long, and F. Zavala. 2024. Vaccines and monoclonal antibodies: New tools for malaria control. Clin. Microbiol. Rev. 37:e0007123. 10.1128/cmr.00071-23 38656211 PMC11237600

[bib52] Murugan, R., S.W. Scally, G. Costa, G. Mustafa, E. Thai, T. Decker, A. Bosch, K. Prieto, E.A. Levashina, J.-P. Julien, and H. Wardemann. 2020. Evolution of protective human antibodies against *Plasmodium falciparum* circumsporozoite protein repeat motifs. Nat. Med. 26:1135–1145. 10.1038/s41591-020-0881-9 32451496

[bib53] Nussenzweig, V., and R.S. Nussenzweig. 1985. Circumsporozoite proteins of malaria parasites. Cell. 42:401–403. 10.1016/0092-8674(85)90093-5 2411417

[bib54] Oyen, D., J.L. Torres, U. Wille-Reece, C.F. Ockenhouse, D. Emerling, J. Glanville, W. Volkmuth, Y. Flores-Garcia, F. Zavala, A.B. Ward, . 2017. Structural basis for antibody recognition of the NANP repeats in *Plasmodium falciparum* circumsporozoite protein. Proc. Natl. Acad. Sci. USA. 114:E10438–E10445. 10.1073/pnas.1715812114 29138320 PMC5715787

[bib55] Phillips, M.A., J.N. Burrows, C. Manyando, R.H. Van Huijsduijnen, W.C. Van Voorhis, and T.N.C. Wells. 2017. Malaria. Nat. Rev. Dis. Primer. 3:17050. 10.1038/nrdp.2017.50 28770814

[bib56] Punjani, A., J.L. Rubinstein, D.J. Fleet, and M.A. Brubaker. 2017. cryoSPARC: algorithms for rapid unsupervised cryo-EM structure determination. Nat. Methods. 14:290–296. 10.1038/nmeth.4169 28165473

[bib57] Reveiz, M., P. Tripathi, L. Da Silva Pereira, P.K. Kiyuka, T. Liu, Y. Yang, B. Zhang, D. Benmohamed, B.G. Bonilla, C.W. Carruthers, . 2025. In silico improvement of affinity for highly protective anti-malarial antibodies. iScience. 28:112903. 10.1016/j.isci.2025.112903 40678545 PMC12269619

[bib58] RTS,S Clinical Trials Partnership . 2015. Efficacy and safety of RTS,S/AS01 malaria vaccine with or without a booster dose in infants and children in Africa: Final results of a phase 3, individually randomised, controlled trial. Lancet Lond. Engl. 386:31–45. 10.1016/S0140-6736(15)60721-8 PMC5626001 25913272

[bib59] Schaefer-Babajew, D., Z. Wang, F. Muecksch, A. Cho, M. Loewe, M. Cipolla, R. Raspe, B. Johnson, M. Canis, J. DaSilva, . 2023. Antibody feedback regulates immune memory after SARS-CoV-2 mRNA vaccination. Nature. 613:735–742. 10.1038/s41586-022-05609-w 36473496 PMC9876794

[bib60] Sok, D., B. Briney, J.G. Jardine, D.W. Kulp, S. Menis, M. Pauthner, A. Wood, E.-C. Lee, K.M. Le, M. Jones, . 2016. Priming HIV-1 broadly neutralizing antibody precursors in human Ig loci transgenic mice. Science. 353:1557–1560. 10.1126/science.aah3945 27608668 PMC5404394

[bib61] Tam, H.H., M.B. Melo, M. Kang, J.M. Pelet, V.M. Ruda, M.H. Foley, J.K. Hu, S. Kumari, J. Crampton, A.D. Baldeon, . 2016. Sustained antigen availability during germinal center initiation enhances antibody responses to vaccination. Proc. Natl. Acad. Sci. USA. 113:E6639–E6648. 10.1073/pnas.1606050113 27702895 PMC5086995

[bib62] Tan, J., L. Piccoli, and A. Lanzavecchia. 2019. The antibody response to *Plasmodium falciparum*: Cues for vaccine design and the discovery of receptor-based antibodies. Annu. Rev. Immunol. 37:225–246. 10.1146/annurev-immunol-042617-053301 30566366

[bib63] Tas, J.M.J., J.-H. Koo, Y.-C. Lin, Z. Xie, J.M. Steichen, A.M. Jackson, B.M. Hauser, X. Wang, C.A. Cottrell, J.L. Torres, . 2022. Antibodies from primary humoral responses modulate the recruitment of naive B cells during secondary responses. Immunity. 55:1856–1871. 10.1016/j.immuni.2022.07.020 35987201 PMC9350677

[bib64] Tian, M., C. Cheng, X. Chen, H. Duan, H.-L. Cheng, M. Dao, Z. Sheng, M. Kimble, L. Wang, S. Lin, . 2016. Induction of HIV neutralizing antibody lineages in mice with diverse precursor repertoires. Cell. 166:1471–1484.e18. 10.1016/j.cell.2016.07.029 27610571 PMC5103708

[bib65] Tripathi, P., M.F. Bender, H. Lei, L. Da Silva Pereira, C.-H. Shen, B. Bonilla, M. Dillon, L. Ou, M. Pancera, L.T. Wang, . 2023. Cryo-EM structures of anti-malarial antibody L9 with circumsporozoite protein reveal trimeric L9 association and complete 27-residue epitope. Structure. 31:480–491.e4. 10.1016/j.str.2023.02.009 36931276 PMC10237622

[bib66] Tripathi, P., J.-H. Koo, X. Chen, L.D. Silva Pereira, M. Dillon, B. Zhang, M. Lofgren, K.T. Nguyen, I.-T. Teng, B. Bonilla, . 2026. Protective immunity against malaria by a nanoparticle CIS43-based junctional vaccine alone or in combination with R21. Npj Vaccin. 11:133. 10.1038/s41541-026-01455-3 PMC13324253 42045246

[bib67] von Boehmer, L., C. Liu, S. Ackerman, A.D. Gitlin, Q. Wang, A. Gazumyan, and M.C. Nussenzweig. 2016. Sequencing and cloning of antigen-specific antibodies from mouse memory B cells. Nat. Protoc. 11:1908–1923. 10.1038/nprot.2016.102 27658009

[bib68] Wang, L.T., N.K. Hurlburt, A. Schön, B.J. Flynn, Y. Flores-Garcia, L.S. Pereira, P.K. Kiyuka, M. Dillon, B. Bonilla, F. Zavala, . 2022. The light chain of the L9 antibody is critical for binding circumsporozoite protein minor repeats and preventing malaria. Cell Rep. 38:110367. 10.1016/j.celrep.2022.110367 35172158 PMC8896312

[bib69] Wang, L.T., A.H. Idris, N.K. Kisalu, P.D. Crompton, and R.A. Seder. 2024. Monoclonal antibodies to the circumsporozoite proteins as an emerging tool for malaria prevention. Nat. Immunol. 25:1530–1545. 10.1038/s41590-024-01938-2 39198635

[bib70] Wang, L.T., L.S. Pereira, Y. Flores-Garcia, J. O’Connor, B.J. Flynn, A. Schön, N.K. Hurlburt, M. Dillon, A.S.P. Yang, A. Fabra-García, . 2020. A potent anti-malarial human monoclonal antibody targets circumsporozoite protein minor repeats and neutralizes sporozoites in the liver. Immunity. 53:733–744.e8. 10.1016/j.immuni.2020.08.014 32946741 PMC7572793

[bib71] Wang, L.T., L.S. Pereira, P.K. Kiyuka, A. Schön, N.K. Kisalu, R. Vistein, M. Dillon, B.G. Bonilla, A. Molina-Cruz, C. Barillas-Mury, . 2021a. Protective effects of combining monoclonal antibodies and vaccines against the *Plasmodium falciparum* circumsporozoite protein. PLoS Pathog. 17:e1010133. 10.1371/journal.ppat.1010133 34871332 PMC8675929

[bib72] Wang, X., R. Ray, S. Kratochvil, E. Melzi, Y.-C. Lin, S. Giguere, L. Xu, J. Warner, D. Cheon, A. Liguori, . 2021b. Multiplexed CRISPR/CAS9-mediated engineering of pre-clinical mouse models bearing native human B cell receptors. EMBO J. 40:e105926. 10.15252/embj.2020105926 33258500 PMC7809789

[bib73] White, M.T., R. Verity, J.T. Griffin, K.P. Asante, S. Owusu-Agyei, B. Greenwood, C. Drakeley, S. Gesase, J. Lusingu, D. Ansong, . 2015. Immunogenicity of the RTS,S/AS01 malaria vaccine and implications for duration of vaccine efficacy: Secondary analysis of data from a phase 3 randomised controlled trial. Lancet Infect. Dis. 15:1450–1458. 10.1016/S1473-3099(15)00239-X 26342424 PMC4655306

[bib74] Williams, K.L., S. Guerrero, Y. Flores-Garcia, D. Kim, K.S. Williamson, C. Siska, P. Smidt, S.Z. Jepson, K. Li, S.M. Dennison, . 2024. A candidate antibody drug for prevention of malaria. Nat. Med. 30:117–129. 10.1038/s41591-023-02659-z 38167935 PMC10803262

[bib75] World Health Organization . 2024. WHO Malaria Policy Advisory Group (MPAG) Meeting Report, 4, 5 and 7 March 2024. World Health Organization, Geneva.

[bib76] Wu, R.L., A.H. Idris, N.M. Berkowitz, M. Happe, M.R. Gaudinski, C. Buettner, L. Strom, S.F. Awan, L.A. Holman, F. Mendoza, . 2022. Low-dose subcutaneous or intravenous monoclonal antibody to prevent malaria. N. Engl. J. Med. 387:397–407. 10.1056/NEJMoa2203067 35921449 PMC9806693

[bib77] Zeeshan, M., M.T. Alam, S. Vinayak, H. Bora, R.K. Tyagi, M.S. Alam, V. Choudhary, P. Mittra, V. Lumb, P.K. Bharti, . 2012. Genetic variation in the *Plasmodium falciparum* circumsporozoite protein in India and its relevance to RTS,S malaria vaccine. PLoS One. 7:e43430. 10.1371/journal.pone.0043430 22912873 PMC3422267

